# The effects of iron deficient and high iron diets on SARS-CoV-2 lung infection and disease

**DOI:** 10.3389/fmicb.2024.1441495

**Published:** 2024-09-04

**Authors:** Agnes Carolin, David Frazer, Kexin Yan, Cameron R. Bishop, Bing Tang, Wilson Nguyen, Sheridan L. Helman, Jay Horvat, Thibaut Larcher, Daniel J. Rawle, Andreas Suhrbier

**Affiliations:** ^1^Inflammation Biology, QIMR Berghofer Medical Research Institute, Brisbane, QLD, Australia; ^2^Molecular Nutrition, QIMR Berghofer Medical Research Institute, Brisbane, QLD, Australia; ^3^School of Biomedical Sciences and Pharmacy, Faculty of Health and Medicine, Hunter Medical Research Institute, University of Newcastle, Callaghan, NSW, Australia; ^4^UMR0703 APEX, INRAE, Oniris, Nantes, France; ^5^GVN Centre of Excellence, Australian Infectious Disease Research Centre, Brisbane, QLD, Australia

**Keywords:** iron deficiency, iron loading, SARS-CoV-2, omicron XBB, C57BL/6J mice, inflammation, lung, RNA-Seq

## Abstract

**Introduction:**

The severity of Coronavirus disease 2019 (COVID-19) caused by the severe acute respiratory syndrome coronavirus 2 (SARS-CoV-2) is often dictated by a range of comorbidities. A considerable literature suggests iron deficiency and iron overload may contribute to increased infection, inflammation and disease severity, although direct causal relationships have been difficult to establish.

**Methods:**

Here we generate iron deficient and iron loaded C57BL/6 J mice by feeding standard low and high iron diets, with mice on a normal iron diet representing controls. All mice were infected with a primary SARS-CoV-2 omicron XBB isolate and lung inflammatory responses were analyzed by histology, immunohistochemistry and RNA-Seq.

**Results:**

Compared with controls, iron deficient mice showed no significant changes in lung viral loads or histopathology, whereas, iron loaded mice showed slightly, but significantly, reduced lung viral loads and histopathology. Transcriptional changes were modest, but illustrated widespread dysregulation of inflammation signatures for both iron deficient vs. controls, and iron loaded vs. controls. Some of these changes could be associated with detrimental outcomes, whereas others would be viewed as beneficial.

**Discussion:**

Diet-associated iron deficiency or overload thus induced modest modulations of inflammatory signatures, but no significant histopathologically detectable disease exacerbations.

## Introduction

In mammals hundreds of proteins use iron in a multitude of cellular activities (Galy et al., 2024) including inflammation and immunity (Ni et al., 2022). Iron levels and distributions in different tissues and cells, under different conditions, are regulated by a complex network of processes (Vogt et al., 2021). Iron studies have been conducted for a range of diseases, with a diverse spectrum of findings (Ali et al., 2017; Charlebois and Pantopoulos, 2023). For instance, for tuberculosis and salmonella, anaemia is linked to poor outcomes, but iron supplementation can exacerbate infections (Hoffmann et al., 2021; Nienaber et al., 2023). For hepatitis B and hepatitis C virus infections, iron overload has been associated with poor prognosis (Schmidt, 2020), whereas for pediatric HIV cases, iron supplementation has been associated with disease progression (Andersen et al., 2022).

The severe acute respiratory syndrome coronavirus 2 (SARS-CoV-2) is the etiological agent of Coronavirus disease 2019 (COVID-19) (Worobey et al., 2022; Crits-Christoph et al., 2023) and has caused a global pandemic involving ~775 million cases and ~ 7 million deaths worldwide (WHO, 2024a). COVID-19 is often associated with a ‘cytokine storm’ and the life threatening, acute respiratory distress syndrome (ARDS). The severity of COVID-19 is influenced by a range of comorbidities (Chenchula et al., 2023; Finnerty et al., 2023; Silaghi-Dumitrescu et al., 2023), with a large body of literature suggesting that iron deficiency and iron overload may also contribute to disease severity (Cavezzi et al., 2020; Campione et al., 2021; Girelli et al., 2021; Habib et al., 2021; Lanser et al., 2021a; Biasiotto and Ferrari, 2022; Gupta et al., 2022; Sonnweber et al., 2022; Zhou et al., 2022; Chaubey et al., 2023; Naidu et al., 2023; Hanson et al., 2024; Liao et al., 2024).

Multiple studies have shown that SARS-CoV-2 infection disrupts iron homeostasis and/or modulates iron-associated biomarkers (Dahan et al., 2020; Taneri et al., 2020; Girelli et al., 2021; Onur et al., 2021; Tojo et al., 2021; Wojciechowska et al., 2021; Bastin et al., 2022; Hegelund et al., 2022; Mohus et al., 2022; Sana and Avneesh, 2022; Aslan et al., 2023; Naidu et al., 2023). This is not unique to SARS-CoV-2, as many infections modulate iron biomarkers (Ward et al., 2022), with clinical determinations of iron status thus generally unreliable in patients presenting with infectious and/or inflammatory diseases (Suchdev et al., 2017; Camaschella, 2019). Establishing the iron status of a patient presenting with COVID-19 is thus similarly complicated. How patients’ iron status prior to SARS-CoV-2 infection affects the severity of COVID-19 after infection is also often difficult to explore in clinical settings, as patients tend to present only after they have developed disease.

Iron deficiency is a widespread problem and is associated with a range of clinical issues, primarily anemia (Gattermann et al., 2021; Pasricha et al., 2021). Pre-existing anemia has been associated with increased mortality risk for hospitalized COVID-19 patients (Lanser et al., 2021a), perhaps due to SARS-CoV-2 infection further exacerbating the anemia (Bergamaschi et al., 2021). Dietary iron deficiency is responsible for about half the ≈2 billion cases of anemia globally (Pasricha et al., 2021; Hussien et al., 2023); however, anemia can have a variety of causes; globally this primarily involves thalassemias, sickle cell trait, and malaria (WHO, 2024b). Other conditions are also associated with anemia, including alcoholism (Manrai et al., 2022), diabetes (Praveen et al., 2020), cardiovascular disease (Lanser et al., 2021b) and chronic obstructive pulmonary disease (Alisamir et al., 2022). Whether the anemia arising from an iron deficient diet, or the comorbidity giving rise to the anemia, is responsible for the increase in COVID-19 severity remains unclear (Ko et al., 2020; Gerayeli et al., 2021; Gregory et al., 2021; Papadopoulos et al., 2021; Andreen et al., 2022). Iron dysregulation and inflammatory stress erythropoiesis have also been associated with long-COVID (Hanson et al., 2024). However, cause and affect are again difficult to verify, as increased iron dysregulation and stress erythropoiesis may be the result of more severe SARS-CoV-2 infections (Bergamaschi et al., 2021; Huerga Encabo et al., 2021), which then predispose to more pronounced long-COVID (Rosa et al., 2023; Zhao et al., 2023).

A number of publications have speculated on a connection between iron overload and increased severity of COVID-19, largely based on iron biomarker studies (Cavezzi et al., 2020; Campione et al., 2021; Habib et al., 2021; Biasiotto and Ferrari, 2022; Gupta et al., 2022; Naidu et al., 2023; Liao et al., 2024). In addition, iron loaded mice, injected with a pseudovirus dis-laying the spike protein, showed an increase in serum CCL4, IL1β, IL-6 and TNFα levels (Chaubey et al., 2023). Iron overload can arise from a number of conditions, most famously hereditary hemochromatosis or thalassemias (Hsu et al., 2022), which lead to a unique pattern of body and cellular iron distributions (Queiroz-Andrade et al., 2009; Cavey et al., 2019). However, iron overload can also arise from excessive dietary iron intake, which is primarily associated with excessive iron supplement consumption (Bell et al., 2000; Deugnier et al., 2002; Barton et al., 2006; Lands and Isang, 2017), but can also be associated with a high iron diet (Kasvosve et al., 2000). Whether pre-existing iron loading due to a high iron diet (in the absence of co-morbidities) exacerbates acute COVID-19 severity remains largely unexplored.

Here we use adult wild-type C57BL/6 J mice and established models of diet-induced iron deficiency and iron overload (Mirciov et al., 2017; Rishi et al., 2018; Ali et al., 2020; Helman et al., 2022), and SARS-CoV-2 infection (Bishop et al., 2024; Carolin et al., 2024). Iron deficient and iron loaded mice were compared with control mice (fed a normal iron diet) after infection with a primary human omicron XBB isolate of SARS-CoV-2 (Stewart et al., 2023). Unlike the original strain isolates, omicron variants effectively utilize the mouse Angiotensin-Converting Enzyme 2 as an entry receptor (Zhang et al., 2022), allowing use of wild-type mice. The impacts of the iron modified diets on viral replication and inflammatory disease in the infected mouse lungs were characterized using histology (H&E staining), immunohistochemistry and RNA-Seq at 2 days post infection (dpi) (peak viral load) and 6 dpi (peak of acute immune pathology) (Bishop et al., 2024; Carolin et al., 2024). Although widespread modulations in transcriptional signatures associated with inflammatory responses were observed, neither iron deficiency nor iron overload resulted in significant increases in viral replication or histopathology.

## Results

### Iron deficient vs. control diet; mouse weights and viral loads

**Figure 1:**
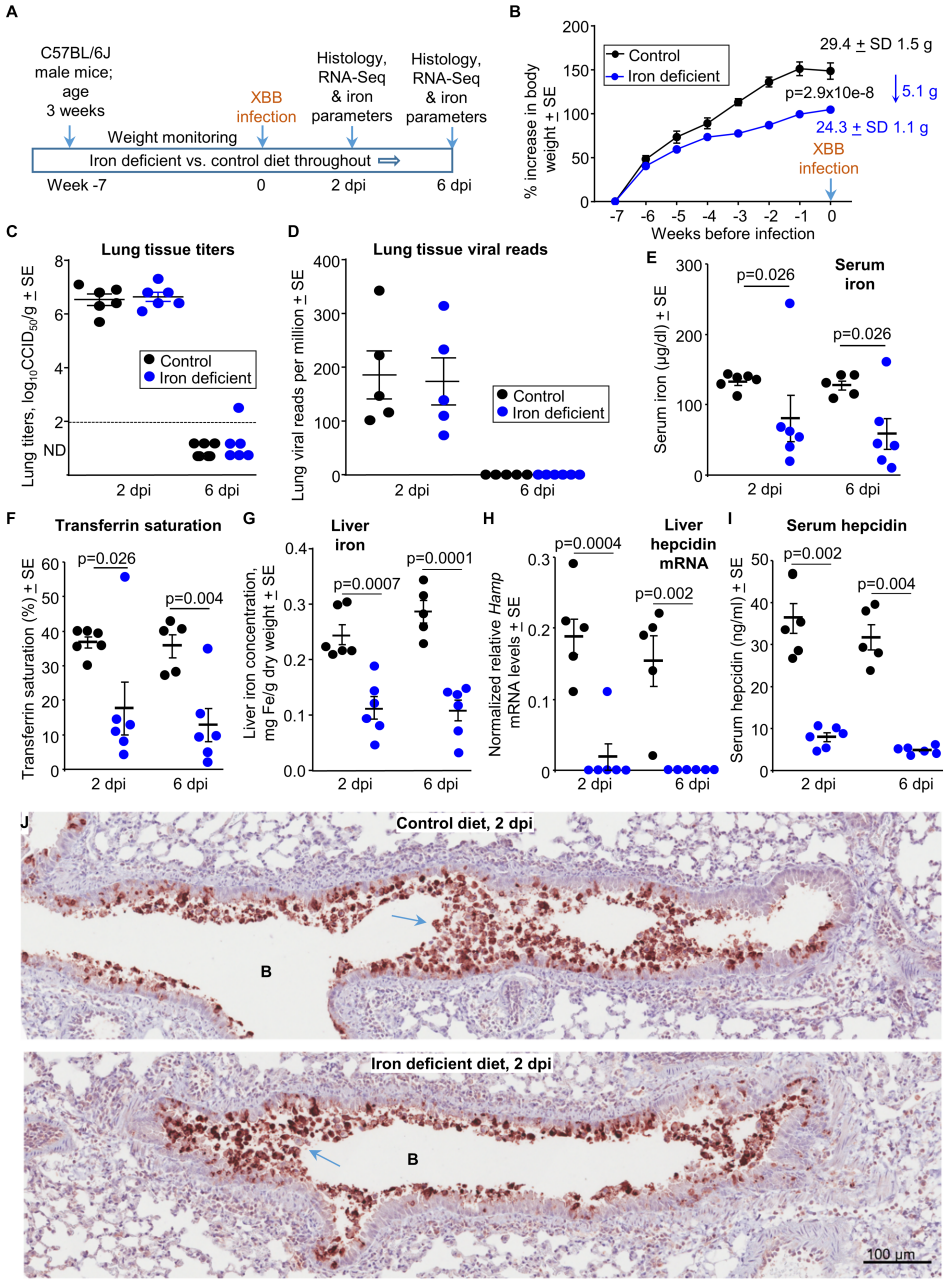
Iron deficient vs. control diets; weights, viral loads and iron parameters. **(A)** Time line of experiment. **(B)** Mean percentage increases in mouse body weights prior to XBB infection. The mean body weights ± SD in grams are also provided for week 0 (also 0 dpi) in text at the top right of the graph. Statistics by *t* test for weight differences at week zero, *n* = 11/12 mice per group. There were no significant weight changes after infection (Supplementary Figure S1). **(C)** Lung tissue XBB virus titers. Limit of detection ≈2 log_10_CCID_50_/g (dashed line); ND, not detected. **(D)** XBB viral read counts obtained from RNA-Seq of lung tissues. **(E–I)** Iron parameters from the same mice described in b-d; Control—black symbols, Iron deficient—blue symbols. **(E)** Serum iron levels. Statistics by Kolmogorov–Smirnov exact tests. **(F)** Transferrin saturation. Statistics by Kolmogorov–Smirnov exact test (2 dpi) and *t* test (6 dpi). **(G)** Liver iron levels. Statistics by *t* tests. **(H)** Liver *Hamp* mRNA levels; the *Hamp* gene encodes hepcidin. Statistics by Kolmogorov–Smirnov exact tests. **(I)** Serum hepcidin levels. Statistics by Kolmogorov–Smirnov exact tests. **(J)** IHC of mouse lung at 2 dpi for mice on the control diet compared with mice on an iron deficient diet. Staining was undertaken using an anti-SARS-CoV-2 spike protein monoclonal antibody. B—bronchial air space. Blue arrows**—**sloughing of virus infected bronchial epithelial cells (and associated cell debris) into the bronchial lumen.

Male C57BL/6 J mice were fed either a control diet (normal iron) or a diet deficient in iron for 7 weeks (Figure 1A). The iron deficient diet resulted in a significant reduction in growth rates so that mice were ≈5 grams (≈17%) lighter than controls just prior to infection (0 dpi) (Figure 1B). XBB infection had no significant effects on the weight of mice in either the control or the iron deficient groups (Supplementary Figure S1). Lungs were harvested on 2 dpi (peak viral titers) and 6 dpi (day of peak lung pathology) (Bishop et al., 2022; Bishop et al., 2024). There were no significant differences in lung tissue titers (Figure 1C), or in lung viral read counts as determined by RNA-Seq (Figure 1D). Viral titers in the nasal turbinates were slightly, but significantly, lower (0.62 log_10_CCID_50_/g, *p* = 0.009) at 2 dpi in mice fed the iron deficient diet (Supplementary Figure 2A).

Thus, although an iron deficient diet moderately reduced the growth of the mice, SARS-CoV-2 tissue titers were not significantly different in lungs, and were slightly lower in nasal turbinates.

### Iron parameters for mice on iron deficient vs. control diets at 2 and 6 dpi

Standard iron parameters were evaluated to confirm the effects of the iron deficient diet. As expected, serum (non-heme) iron levels (Figure 1E) and transferrin saturation levels (Figure 1F) were significantly lower in mice on the iron deficient diet. We could not identify any reasons for high serum iron and transferrin saturation levels in two iron deficient mice (Figures 1E,F, one at 2 dpi and one at 6 dpi), with results confirmed by repeat assays. They perhaps reiterate the unreliable nature of such iron studies during active inflammatory disease (Suchdev et al., 2017; Camaschella, 2019). Mice on the iron deficient diet showed significantly lower liver iron levels (Figure 1G), with assessments of liver iron arguably the most reliable method (Kohgo et al., 2008) to confirm that the diet had successfully reduced iron levels. Liver hepcidin mRNA levels and serum hepcidin levels were also significantly lower in mice on the iron deficient diet (Figures 1H,I), consisted with the role of hepcidin in iron sequestration (Ginzburg, 2019). Although inflammation can up-regulate hepcidin, the robust iron deficiency likely plays the dominant role in suppressing hepcidin levels in this setting (Figure 1I) (Darshan et al., 2010).

In summary, taken together, the measured iron parameters confirmed that the iron deficient diet had successfully reduced iron levels in the mice.

### Iron deficient vs. control diet; immunohistochemistry at 2 dpi

Lung sections were stained with a SARS-CoV-2 spike specific monoclonal antibody (Morgan et al., 2023). Staining was primarily associated with the bronchial epithelium and cellular debris in the bronchial lumen (Figure 1J). The latter likely represents bronchial epithelial cells sloughed-off into the airways after infection-induced cytopathic effects (CPE). No overt differences in staining was observed for mice on the iron deficient vs. control diets, consistent with data in Figures 1C,D. IHC staining of an uninfected mouse lung, illustrating the low level of background staining, is shown in Supplementary Figure S3A.

### Iron deficient vs. control diet; histochemistry and histopathology

**Figure 2:**
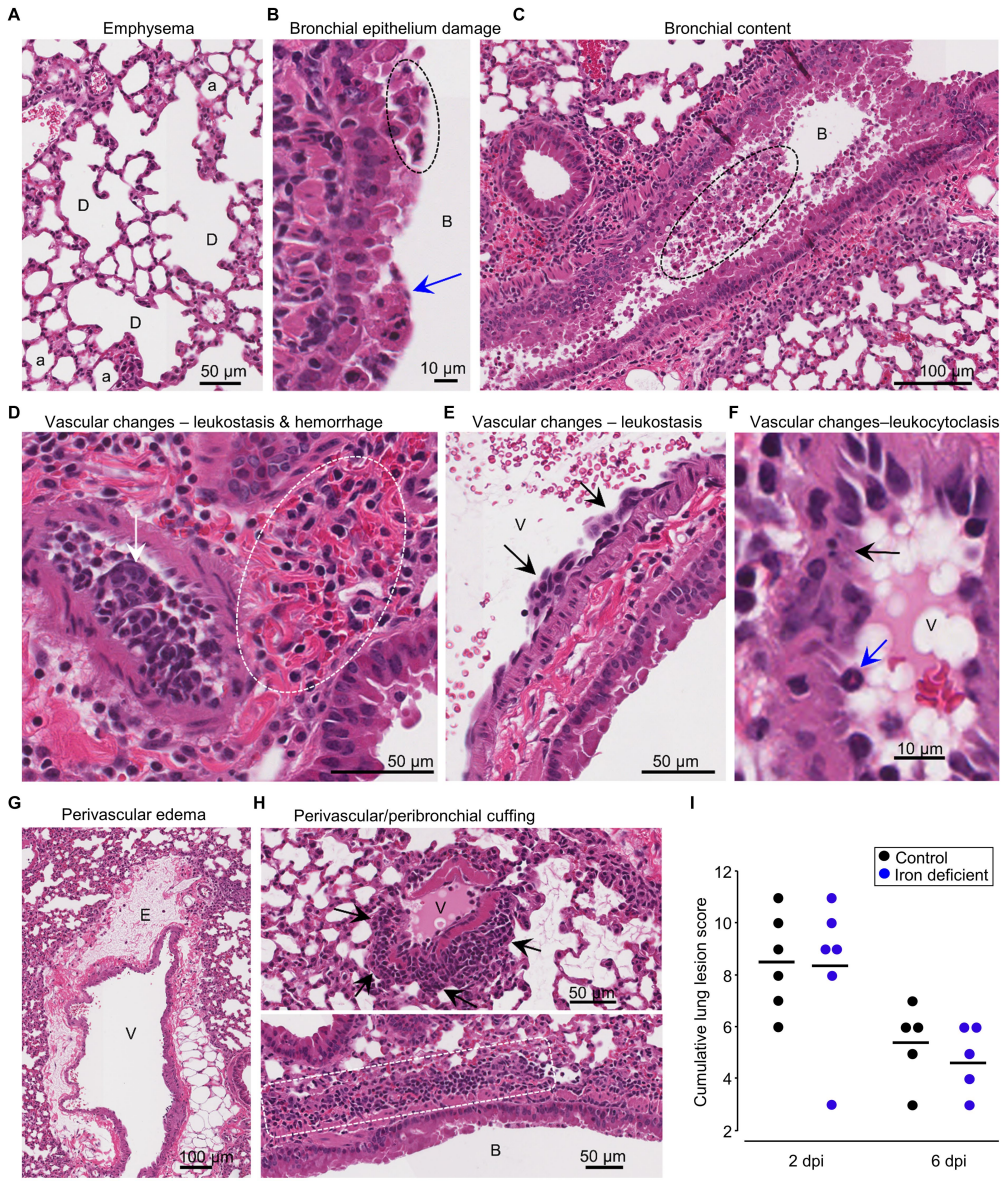
Iron deficient vs. control diets; lung histopathology. H&E staining of infected lungs. **(A)** Iron deficient diet 2 dpi showing dilated coalescing alveoli (D) illustrating emphysema. a, alveoli. Whole slide score for emphysema = 1 on a 0–2 scale. **(B)** Control diet 2 dpi showing necrotic bronchial epithelia cells (blue arrow) and partial loss of bronchial epithelial architecture (dashed oval). B, bronchial lumen. Whole slide score for bronchial epithelial damage = 2 on a 0–3 scale. **(C)** Control diet 2 dpi showing cellular debris in bronchial air space (dashed oval); this material stains positive for viral antigen. B, bronchial air space. Whole slide score for bronchial content = 2 on a 0–3 scale. **(D)** Control diet 2 dpi showing occlusion of the vascular lumen by clumps of leukocytes (leukostasis) (white arrow), with perivascular hemorrhage (dashed oval). Whole slide score for vascular wall changes = 3 on a 0–3 scale. **(E)** Iron deficient diet 6 dpi showing clumps of leukocytes adherent to the blood vessel intima (arrows). V, vascular lumen. Whole slide score for vascular wall changes = 2. **(F)** Control diet 2 dpi showing leukocytoclasis (vascular damage caused by nuclear debris from infiltrating neutrophils) (black arrow). Blue arrow—neutrophil. Whole slide score for vascular wall changes = 3. **(G)** Control diet 2 dpi showing perivascular edema **(E)**. V, vascular lumen. Whole slide score for edema = 2 on a 0–2 scale. **(H)** (Top) Iron deficient diet 2 dpi showing perivascular cuffing (arrows). V, vascular lumen. Whole slide score for perivascular/peribronchial cuffing =2. (Bottom) Control diet showing peribronchial cuffing (white dashed box showing leukocytes). B, bronchial lumen. Whole slide score for perivascular/peribronchial cuffing =3 on a 0–3 scale. **(I)** Cumulative lung lesion scores for each mouse; raw data is shown in Supplementary Figure S4A.

Lung sections taken at 2 and 6 dpi were stained with H&E and scanned slides were examined by a European board-certified veterinary pathologist for histopathological lesions. Lung lesions were scored using 6 criteria with examples shown; (i) emphysema (Figure 2A), (ii) bronchial epithelium damage (Figure 2B), (iii) bronchial content (Figure 2C), which occasionally included red blood cells (RBC) (Supplementary Figure S3B), (iv) vascular changes comprising leukostasis (Figures 2D,E), perivascular hemorrhage (Figure 2D) and/or leukocytoclasis (Figure 2F), (v) perivascular edema (Figure 2G) and (vi) perivascular and/or peribronchial cuffing (Figure 2H). Scores were summed to provide a cumulative score for each mouse (Supplementary Figure S4A), with no significant differences emerging between groups (Figure 2I).

White space analysis, an approximate measure of lung consolidation, showed the expected (Rawle et al., 2021; Yan et al., 2022; Dumenil et al., 2023) significant reduction in virus-infected mice compared with uninfected mice (Supplementary Figure S5). However, no significant differences emerged between mice on the different diets, although white space reductions appeared to have occurred slightly earlier (2 dpi) in some iron deficient mice (Supplementary Figure S5).

Using the same H&E stained sections, a pixel count analysis was undertaken to generate a ratio of nuclear (purple) to cytoplasmic (red) staining, which provides an approximate measure of leukocyte infiltration (Prow et al., 2019; Dumenil et al., 2023). As expected, infected mice showed significantly more infiltrates than naïve mice; however, no significant differences emerged between infected mice on the different diets (Supplementary Figures S6A,B).

### Iron deficient vs. control diet; lung RNA-Seq at 2 dpi

**Figure 3:**
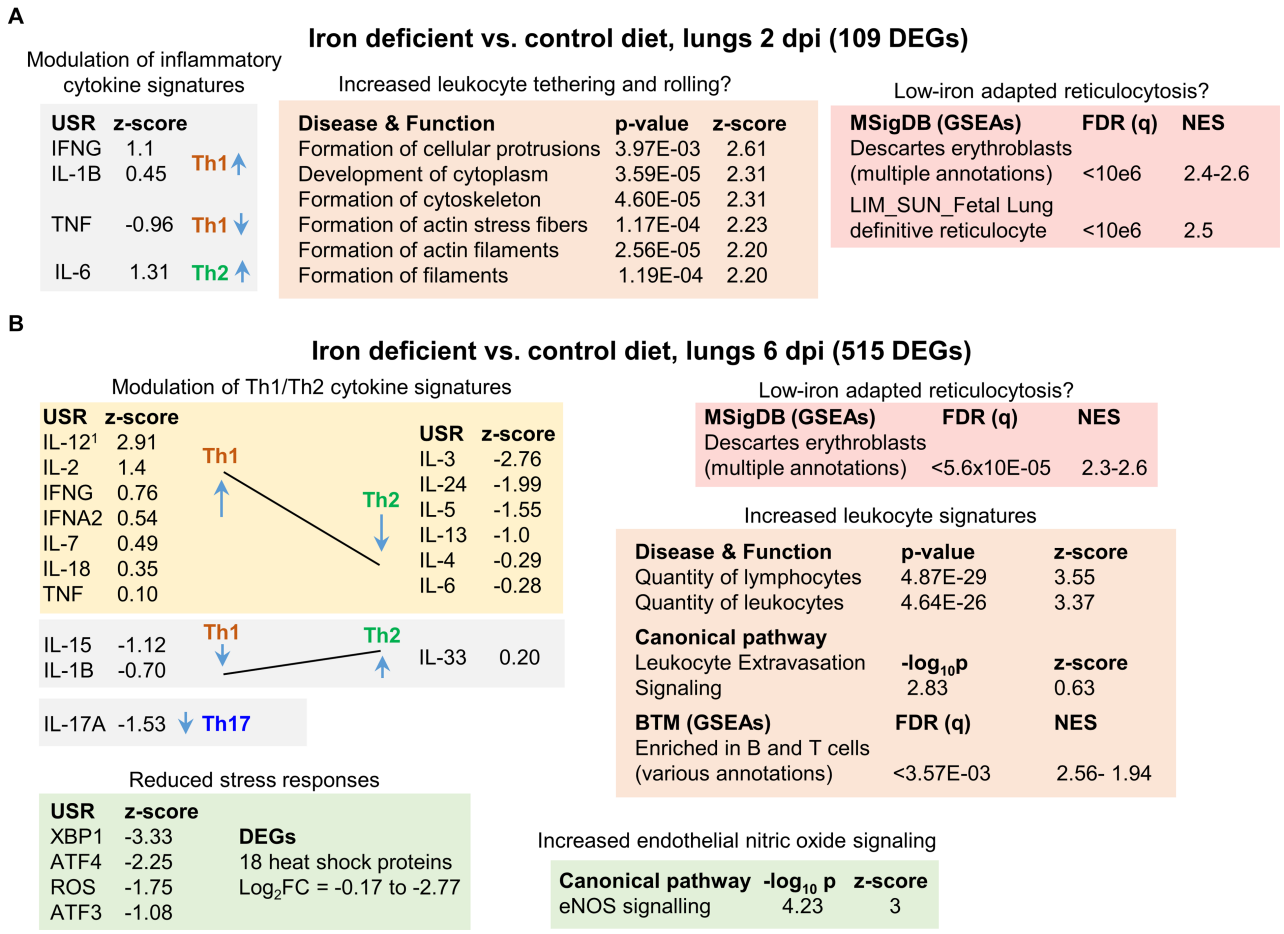
Iron deficient vs. control diets; bioinformatic summary. **(A)** RNA-Seq analysis of lungs from mice fed an iron deficient vs. control diet at 2 dpi yielded 109 DEGs. DEGs were analyzed by IPA using the Up-Steam Regulator (USR) (cytokine annotations shown), and Disease and Function features. The All genes list was interrogated using GSEAs and gene sets provided by MSigDB. Full data sets are provided in Supplementary Table S1. ^1^USR annotation is “IL-12 (family)” and Molecular Type is “group” rather than “cytokine.” **(B)** RNA-Seq analysis of lungs from mice fed an iron deficient vs. control diet at 6 dpi yielded 515 DEGs. Bioinformatic summary as for a, with the addition of USRs associated with stress responses, and IPA Canonical pathway annotations. Full data sets are provided in Supplementary Table S2.

RNA-Seq analysis of lungs at 2 dpi from mice fed an iron deficient vs. a control diet identified only 109 DEGs, with all but 5 of these showing low (<1 log_2_) fold change (Supplementary Table S1). (PC2/PC1 plots for all the RNA-Seq data are shown in Supplementary Figure S7). A heat map of the top 100 genes that provided the greatest contribution to the segregation between iron deficient vs. control groups, further illustrated that gene expression differences between these groups was low and not particularly focused on any specific set of genes (Supplementary Figures S8A,B). The results from the bioinformatic analyses are shown in Supplementary Table S1 and are summarized in Figure 3A.

The 109 DEGs were analyzed by Ingenuity Pathway Analysis (IPA). Using the Up Stream Regulator (USR) feature, mild modulation of inflammatory cytokine signatures was identified (Figure 3A). As iron deficiency has been associated with changes in the Th1/Th2 cytokine balance (Roth-Walter et al., 2017; Ni et al., 2022; Roth-Walter, 2022), the Th1 or Th2 association for each cytokine is shown (Figure 3A), although 2 dpi is generally too early for significant adaptive T cell responses. The IPA Diseases or Functions feature identified a series of top annotations associated with cellular protrusions, cytoskeleton and actin (Figure 3A). These annotations may indicate increased leukocyte tethering and rolling (Kameritsch and Renkawitz, 2020) in the iron deficient mice. However, significant histological differences were not apparent (Supplementary Figure S9), as might be expected with only 109 DEGs.

Gene set enrichment analyses (GSEAs) were undertaken using the ‘All genes’ list (ranked by fold change) (Supplementary Table S1) and gene sets from the Molecular Signatures Database (MSigDB) (Liberzon et al., 2015; Castanza et al., 2023). This analysis identified a series of annotations, with high Normalized Enrichment Scores (NES), that were associated with erythroblasts (Figure 3A, NES ≈ 2.4–2.6). Erythroblasts are ordinarily restricted to bone marrow, with the loss of the nucleus from these cells preceding release of the resulting reticulocytes, immature red blood cells (RBC), into the circulation. Reticulocytes retain the mRNA profile of erythroblasts in the final stages of maturation (Goh et al., 2007). These erythroblast annotations thus likely reflect an increase in gene signatures associated with reticulocytosis in iron deficient mice. Viral infections, including COVID-19, can result in significant damage to RBC (Al-Kuraishy et al., 2022; Russo et al., 2022), with COVID-19 also able to infect RBC progenitors (Kronstein-Wiedemann et al., 2022). Erythropoiesis is thus stimulated (Hanson et al., 2024), with erythroblasts adapting to iron deficient conditions by modulating gene expression (Liu et al., 2008; Wollmann et al., 2014; Kobayashi et al., 2017), with such modulation likely giving rise to these erythroblast annotations.

In summary, at 2 dpi iron deficiency imparted only mild transcriptional changes, which were associated with minor modulation of inflammatory cytokine, and perhaps tethering and rolling, signatures. Erythroblast/reticulocyte signatures also indicated erythroblast adaptation to low iron conditions.

### Iron deficient vs. control diet; lung RNA-Seq at 6 dpi

RNA-Seq analysis of lungs at 6 dpi from mice fed an iron deficient diet vs. a control diet identified 515 DEGs, with all but 23 of these showing low (<1 log_2_) fold change (Supplementary Table S2). The results from the bioinformatic analyses are shown in Supplementary Table S2 and are summarized in Figure 3B (see below).

IPA USR analysis again identified modulation of cytokine response signatures that were generally associated with up-regulation of Th1 signatures and down-regulation of Th2 signatures (Figure 3B). This contrasts with previous reports that suggest immune activation under iron-deficient conditions results in the expansion of Th2, but not Th1 cells (Roth-Walter et al., 2017; Roth-Walter, 2022). However, such Th1/Th2 modulation is likely to be setting dependent (Ni et al., 2022). For instance, iron deficiency is reported to blunt IL-6 responses (Ekiz et al., 2005; Darshan et al., 2010) in some settings, but not others (Nakagawa et al., 2014). Consistent with the observations herein (Figure 3B), iron deficiency has been reported to reduce IL-4 (Kuvibidila et al., 2012) and IL-17A responses (Li et al., 2021; Teh et al., 2021). In addition, iron deficiency has been reported to cause non-proliferating, altruistic T cells to produce IL-2 (Berg et al., 2020).

Top IPA Diseases or Functions annotations indicated an increase in the quantity of lymphocytes/leukocytes in infected iron-deficient mouse lungs (Figure 3B; Increased leukocyte signatures). BTM GSEAs suggest these increases are primarily associated with B cells (Supplementary Table S2), consistent with identification of CXCR5 as an upregulated DEG (log_2_FC = 0.88) (Supplementary Table S2). CXCR5 is the receptor for CXCL13, which is the key chemokine for B cell recruitment to sites of inflammation (Kowarik et al., 2012; Armas-González et al., 2018). However, histological analyses indicated only marginal, non-significant, increases in leukocytes in iron deficient mice (Supplementary Figure S6A, 6 dpi; Supplementary Figure S4A, cuffing 6 dpi), arguing the transcriptional modulations (Figure 3B) do not reflect an overall overt increase in inflammatory infiltrates. Instead, they may reflect transcription changes associated with (i) modest increases in infiltrates, with, for instance, the extravasation annotation indicating a relatively low z-score (Figure 3B, Z-score = 0.63), and/or (ii) Th1/Th2 modulation changing leukocyte transcriptional profiles, and/or (iii) changes in the type of cells infiltrating the infected lungs in iron deficient mice (see below).

The top upregulated Canonical pathway was endothelial nitric oxide synthase (Figure 3B, eNOS), with increased endothelial nitric oxide (NO) signaling previously associated with iron deficiency (Dunaway et al., 2023). eNOS has a central role in endothelial homeostasis and is generally viewed as serving a beneficial role in lung inflammation (Qi et al., 2016; Guimarães et al., 2021; Ren et al., 2021) and ARDS (Albertine et al., 1999; Vassiliou et al., 2021). eNOS uncoupling can lead to generation of reactive oxygen species (ROS) and lung injury (Gross et al., 2015). However, this was not indicated in this setting as the ROS signature was reduced in iron deficient mice (Figure 3B, ROS). A number of other stress responses signatures were also lower in iron deficient mice; specifically, XBP1 (endoplasmic reticulum stress) (Cohen et al., 2017), ATF3 and ATF4 (stress-induced transcription factors) (Bradley et al., 2021; Shahriari-Felordi et al., 2022; Li et al., 2023; Niethamer et al., 2023) (Figure 3B). In addition, expression of 18 heat shock protein mRNAs was lower (Figure 3B, DEGs), with Hspa1a and Hspa1b (Hsp70 family members) (Bishop et al., 2024) the most down-regulated DEGs (Supplementary Table S2).

In summary, iron deficient mice at 6 dpi showed modest transcriptional changes when compared to controls. Bioinformatic analyses indicated signatures associated with increased Th1/Th2 ratios, modulated leukocyte expression patterns, and elevated eNOS and reduced stress responses.

### Iron loading vs. control diet; weights and lung viral loads

**Figure 4:**
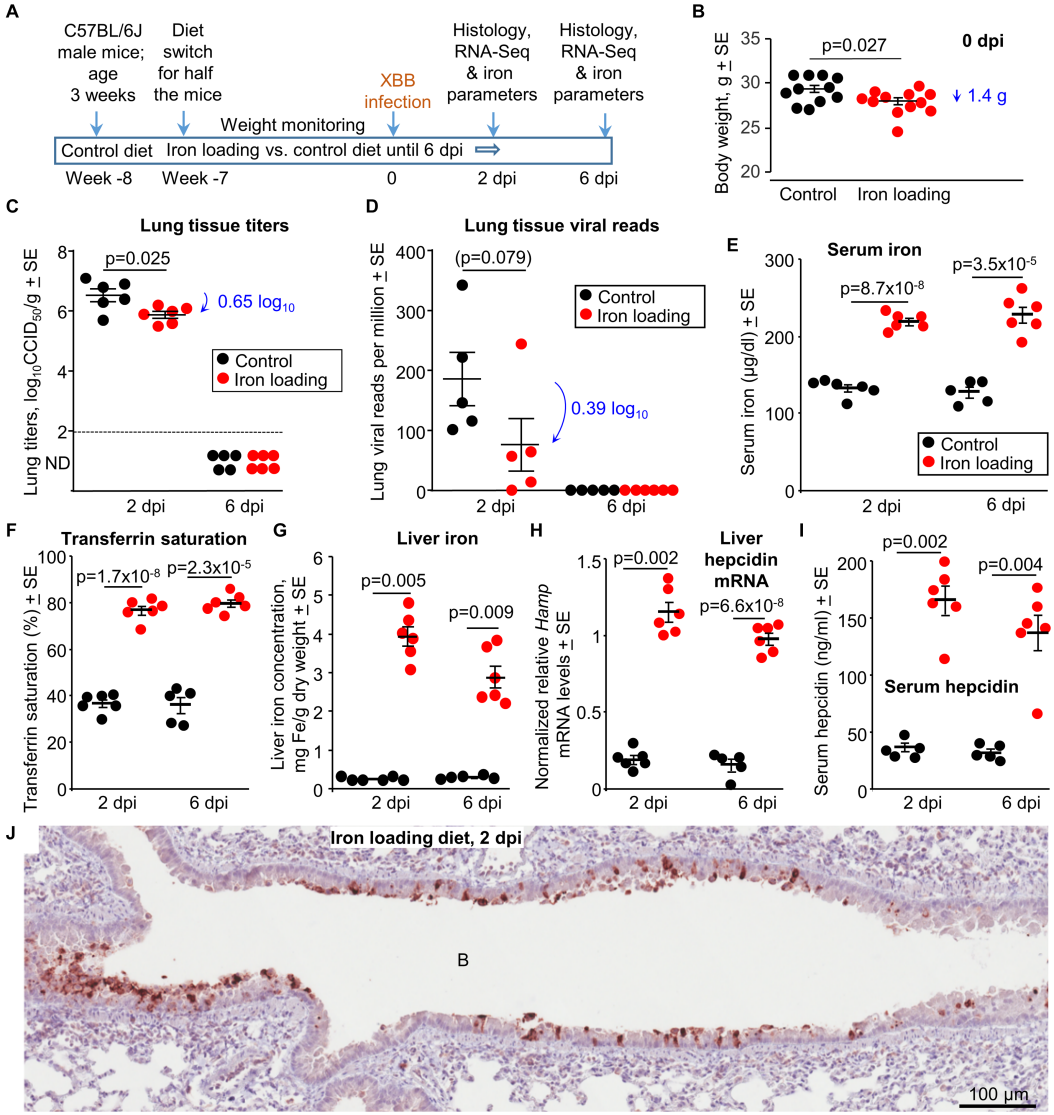
Iron loading vs. control diets; weights, viral loads and iron prarameters. **(A)** Time line of experiment. **(B)** Mouse body weights at 0 dpi, prior to XBB infection. Statistics by *t* test, *n* = 11/12 mice per group. There were no significant weight changes after infection (Supplementary Figure S1). **(C)** Lung tissue XBB virus titers. Limit of detection ≈2 log_10_CCID_50_/g (dashed line); ND – not detected. **(D)** XBB viral read counts obtained from RNA-Seq of lung tissues. **(E–I)** Iron parameters from the same mice described in b-d; Controls—black symbols, Iron loaded—red symbols. **(E)** Serum iron levels. Statistics by *t* tests. **(F)** Transferrin saturation. Statistics by *t* tests. **(G)** Liver iron levels. Statistics by Kolmogorov–Smirnov exact tests. **(H)** Liver *Hamp* mRNA levels. Statistics by Kolmogorov–Smirnov exact test (2 dpi) and *t* test (6 dpi). **(I)** Serum hepcidin levels. Statistics by Kolmogorov–Smirnov exact tests. (Data for control mice in b-i is the same as in Figure 1). **(J)** IHC of lung at 2 dpi for a mouse on the iron loading diet, using an anti-SARS-CoV-2 spike protein monoclonal antibody. B, bronchial air space. IHC of lung from a control mouse is shown in Figure 1J (top image). Staining of an uninfected lung is shown in Supplementary Figure S3A.

Male C57BL/6 J mice were fed either a control diet or an iron loading diet for 7 weeks starting at 4 weeks of age (Figure 4A). The iron loading diet resulted in a small but significant reduction in body weight (mean 1.4 g reduction) at 0 dpi (Figure 4B). No significant weight changes were observed post XBB infection in either the control or the iron loading groups (Supplementary Figure S1). Lung tissue titers at 2 dpi showed a modest, but significant, 0.65 log_10_CCID_50_/g reduction in lung viral titers from mice fed the iron loading diet (Figure 4C). Nasal turbinate viral titers showed no significant differences at 2 dpi (Supplementary Figure S2B). Viral lung read counts (from RNA-Seq analysis) also showed a reduction (of 0.39 log_10_), but this did not reach significance (Figure 4D).

Thus, the iron loading diet marginally reduced the mean mouse body weight, with lungs showing modest, but significant, viral titer reductions at 2 dpi. The latter is consistent with some (Singh et al., 2023), but not other (Chaubey et al., 2023), *in vitro* studies.

### Iron parameters for mice on iron loading vs. control diets at 2 and 6 dpi

Standard iron parameters were again evaluated to confirm the effects of the iron loading diet. As expected, serum iron levels (Figure 4E) and transferrin saturation levels (Figure 4F) were significantly higher in mice on the iron loading diet. Mice on the iron loading diet showed significantly higher liver iron levels (Figure 4G). mRNA levels for liver *Hamp* (the gene that codes for hepcidin) (Figure 4H) and serum hepcidin protein levels (Figure 4I) were also significantly higher in mice on the iron loading diet.

In summary, the iron parameters all confirmed that the iron loading diet had successfully increased iron levels in the mice.

### Iron loading vs. control diet; immunohistochemistry at 2 dpi

Lung sections were stained with a SARS-CoV-2 specific monoclonal antibody. Staining was primarily associated with the bronchial epithelium, with minimal stained material in the bronchial airways (Figure 4J). Staining was less abundant (compared with controls, Figure 1J), consistent with the lower viral load (Figure 4C). IHC staining of an uninfected mouse lung is shown in Supplementary Figure S3A.

### Iron loading vs. control diet; histochemistry and lung lesions

**Figure 5:**
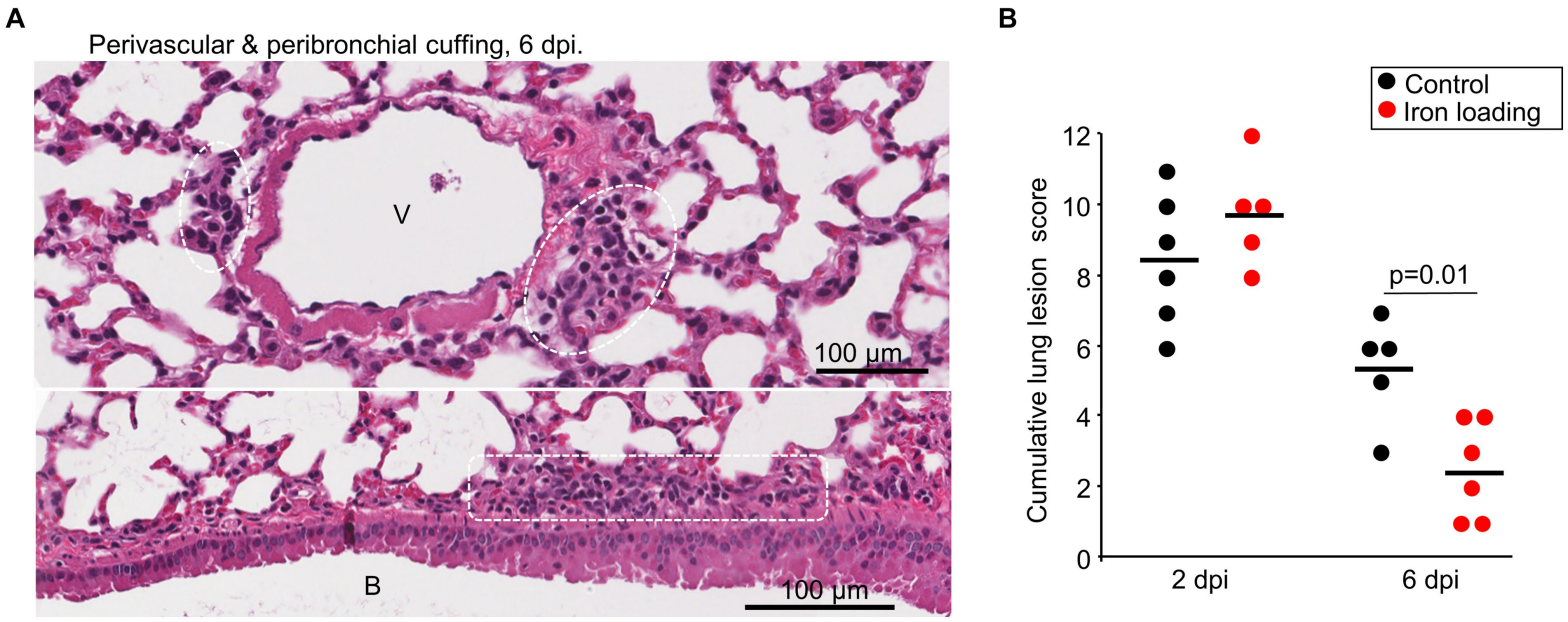
Iron loading vs. control diets; histology. **(A)** Example of perivascular cuffing (top) and peribronchial cuffing (bottom) at 6 dpi for iron loaded mice. Leukocytes indicated by white dashed box/ovals. V, vascular lumen. B, bronchial lumen. Whole slide score for perivascular/peribronchial cuffing =1 on a 0–3 scale. **(B)** Cumulative lung lesion scores for each mouse, raw data is shown in Supplementary Figure S4B. Statistics by *t* test.

Lung sections taken at 2 and 6 dpi were stained by H&E and scanned slides were examined by a veterinary pathologist. Lung lesions were scored as above, with lesions at 6 dpi emerging to be slightly less severe across the scoring criteria (Supplementary Figure S4B, Figure 5A), with the cumulative lesion score significantly lower for iron loaded mice when compared with mice on the control diet (Figure 5B). This observation likely reflects the lower viral loads in lungs from iron loaded mice (Figure 4C).

White space analysis (Supplementary Figure S10) and ratios of nuclear (purple) to cytoplasmic (red) staining (Supplementary Figure S6C) showed no significant differences between infected mice on iron loading vs. control diets.

### Iron loading vs. control diet; lung RNA-Seq at 2 dpi

**Figure 6:**
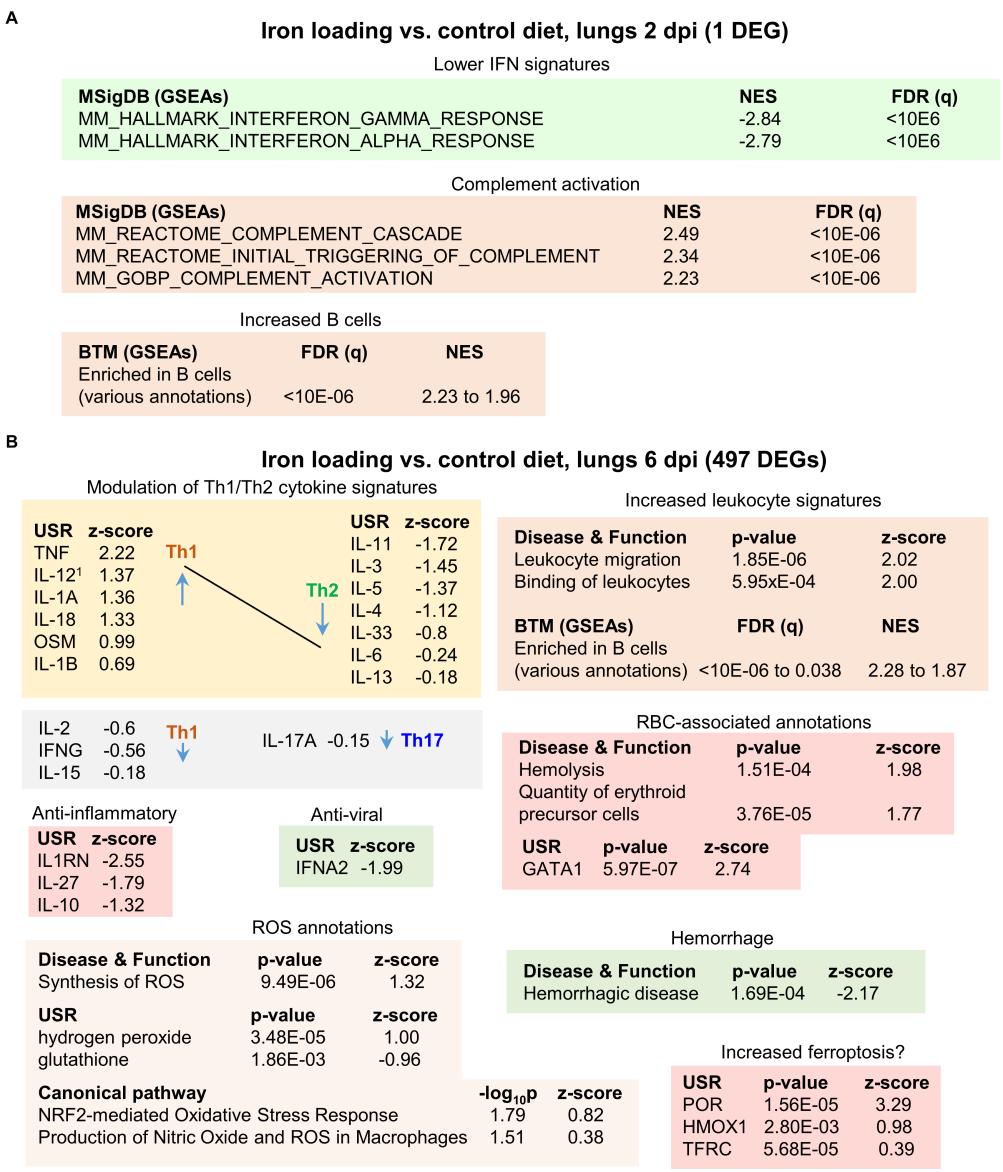
Iron loading vs. control diets; bioinformatic summary. **(A)** RNA-Seq analysis of lungs from mice fed an iron loading vs. control diet at 2 dpi yielded only 1 DEG, insufficient for pathway analysis. The ‘All genes’ list was interrogated using GSEAs and the gene sets provided by MSigDB. Full data sets are available in Supplementary Table S3. **(B)** RNA-Seq analysis of lungs from mice fed an iron loading vs. control diet at 6 dpi yielded 497 DEGs. DEGs were analyzed by IPA as in Figure 3. Full data sets are available in Supplementary Table S4.

RNA-Seq analysis of lungs at 2 dpi from mice fed an iron loading vs. control diet identified only 1 DEG, insufficient for meaningful pathway analysis. Full gene lists and bioinformatic analyses are provided in Supplementary Table S3 and are summarized in Figure 6A (see below).

GSEAs using the MSigDB gene sets provided a number of IFN annotations with high negative NES values (Figure 6A). These likely reflect the lower lung viral loads (Figures 4C,D), with less virus replication in iron loaded mice resulting in less stimulation of IFN responses. MSigDB GSEAs also identified complement activation with high positive NES values (Figure 6A). SARS-CoV2 is known to activate complement via the lectin (Ali et al., 2024) and alternative pathways (Yu et al., 2020). Complement activation can mediate antiviral effects against SARS-CoV-2 (Santiesteban-Lores et al., 2021), providing a potential explanation for the reduction in viral loads. Iron infusions are reported to trigger complement via the lectin and alternative pathways (Hempel et al., 2016; Faria et al., 2019). One might thus speculate that iron loading reduces the threshold for complement activation during SARS-CoV-2 infection. The one DEGs (Per1) may also be linked to complement activation (Shivshankar et al., 2020). However, it should be noted that complement activation primarily involves proteolytic processes that are not directly detectable by RNA-Seq. Complement activation by adaptive immune responses (mediated by IgM/IgG and the classical pathway) comes later during the course of infection, and can also (Ali et al., 2024) contribute to COVID-19 severity (Georg et al., 2022). However, complement activation was not identified in iron loaded mice at 6 dpi (Supplementary Table S4), with virus largely cleared in the current model at this time (Figures 4C,D).

GSEAs using the BTM gene sets suggested an increased number of B cells infiltrating the infected lungs in iron loaded mice (Figure 6A).

### Iron loading vs. control diet; lung RNA-Seq at 6 dpi

RNA-Seq analysis of lungs at 6 dpi from mice fed an iron loading vs. a control diet identified 497 DEGs. Fold changes were again modest, with only 2 genes showing a log_2_ fold change >2. Full gene lists and bioinformatics are provided in Supplementary Table S4 and are summarized in Figure 6B.

Modulation of Th1/Th2 cytokines was again apparent with mostly increased Th1 and reduced Th2 USR cytokine z scores in mice on an iron loading diet (Figure 6B). This result contrasts with *Salmonella typhimurium* infection of mice on a high iron diet where Th1 responses were inhibited (Pfeifhofer-Obermair et al., 2021). However, the effects of high iron on the Th1/Th2 balance may be setting specific, with, for instance, iron promoting M1 differentiation of macrophages (Ni et al., 2022), and lung macrophages playing a central role in COVID-19 inflammation (Olivier et al., 2023). Several anti-inflammatory USR cytokine signatures provided negative z-scores (Figure 6B), indicating less anti-inflammatory activity in iron loaded mice. However, the IFNA2 USR signature had a negative z-score (Figure 6B, Anti-viral), suggesting reduced inflammation, with type I IFNs major drivers of inflammation (Ji et al., 2023). A reduced type I IFN signature is consistent with the lower viral loads at 2 dpi (Figure 4C) and the negative NES for IFN-associated GSEAs at 2 dpi (Figure 6A).

The annotations associated with increased leukocyte signatures (Figure 6B) do not reflect significant, histologically observable, increases in leukocytes infiltrates (Figure 5A, Supplementary Figures S4B, S6C). This again suggests they are associated with modest infiltrate changes, Th1/Th2 response changes and/or changes in the cell types infiltrating the lungs (see below). A series of BTMs suggested an increase in B cells (Figure 6B), although no chemokines or chemokine receptors that would readily explain migration of B cells into the lungs were present in the DEG list (Supplementary Table S4).

Iron has often been associated with ROS production (Chen et al., 2020; Nousis et al., 2023), and ROS-associated annotations were identified amongst the Canonical pathway annotations. However, z-scores were modest (≤1.3) (Figure 6B, ROS annotations), perhaps ameliorated by the lower viral loads. Increased ROS in iron overload setting is often ascribe to the Fenton reaction (e.g., (Mizumura and Gon, 2021)); however, the physiological relevance of this reaction *in vivo* is not without controversy (Muranov, 2024).

The negative z-score for the hemorrhagic disease annotation (Figure 6B) likely reflects the reductions in viral load, with lung hemorrhage well documented in COVID-19 mouse models (Sun et al., 2020; Ali et al., 2024). A number of transcription factor USRs also showed negative z-scores in iron loaded mice (Supplementary Table S4). These include, FOXC1 (Ahmed et al., 2022), XBP1 (Fernández et al., 2024a), EIF4E (Korneeva et al., 2023) and SREBF1 (aka SREBP1) (Soares et al., 2023), which are induced by SARS-CoV-2 infection. FOXC1 (Ma et al., 2020), XBP1 (Wang et al., 2023), and EIF4E (Schwarz et al., 2002), as well as TCF3 (Miao et al., 2014), are also involved in wound repair. Reduced infection (Figures 4C,D) and/or an ensuing reduced requirement for tissue repair, may explain these negative z-scores.

A number of RBC-associated annotations were identified, including hemolysis (Figure 6B). Although viral infections (Russo et al., 2022), including SARS-CoV-2 (Al-Kuraishy et al., 2022), can cause hemolysis, why this should be higher in iron loaded mice is unclear. This may be due to the toxic effects of iron on RBC (Kozlova et al., 2022), or is associated with complement activation (Figure 6A), with complement-mediated hemolysis a well-documented phenomenon (Bortolotti et al., 2023; Xiao et al., 2024). GATA1 is the master regulator of erythropoiesis (Gutiérrez et al., 2020), which might be upregulated to compensate for RBC loss.

POR (NADPH-cytochrome P450 oxidoreductase) was identified as the top scoring USR by z-score (Figure 6B, Supplementary Table S4). Amongst other functions, POR is involved in the induction of ferroptosis (Ai et al., 2021). Ferroptosis is a form of cell death promoted by iron that involves peroxidation of lipids (Zhang et al., 2023), which has been implicated in tissue damage during COVID-19 (Jacobs et al., 2020; Qiu et al., 2024). Heme oxygenase 1(HMOX1) is a crucial ferroptosis factor (Chen et al., 2023), and the transferrin receptor (TFRC) is a ferroptosis marker (Feng et al., 2020), with increased ROS/H_2_0_2_ and reduced glutathione (Figure 6B) crucial to the process of lipid peroxidation (Zhang et al., 2023). However, it should be noted that there is no single universal ferroptosis pathway, with many initiators, sensitizers and modulators (Dixon and Pratt, 2023); hence reliable ferroptosis annotations are often lacking in bioinformatic pathway tools such as IPA.

In summary, iron loaded mice showed modestly lower lung viral loads at 2 dpi, with RNA-Seq indicating modest transcriptional changes. Signatures at 6 dpi in iron loaded mice were associated with a general bias toward increased Th1/Th2 cytokine ratios, hemolysis, ROS and perhaps ferroptosis, but also reduced type I IFN and hemorrhage.

### Dysregulated cell compositions in infected lungs of iron deficient and iron loaded mice

To gain insights into how iron deficiency and iron overload might influence the cellular compositions in the lungs after SARS-CoV-2 infection, cellular deconvolution (SpatialDecon) analysis was undertaken. This used the normalized count matrices (that provide the number of aligned reads for each gene for each mouse) and the gene expression matrices from the Lung mouse cell atlas.

**Figure 7:**
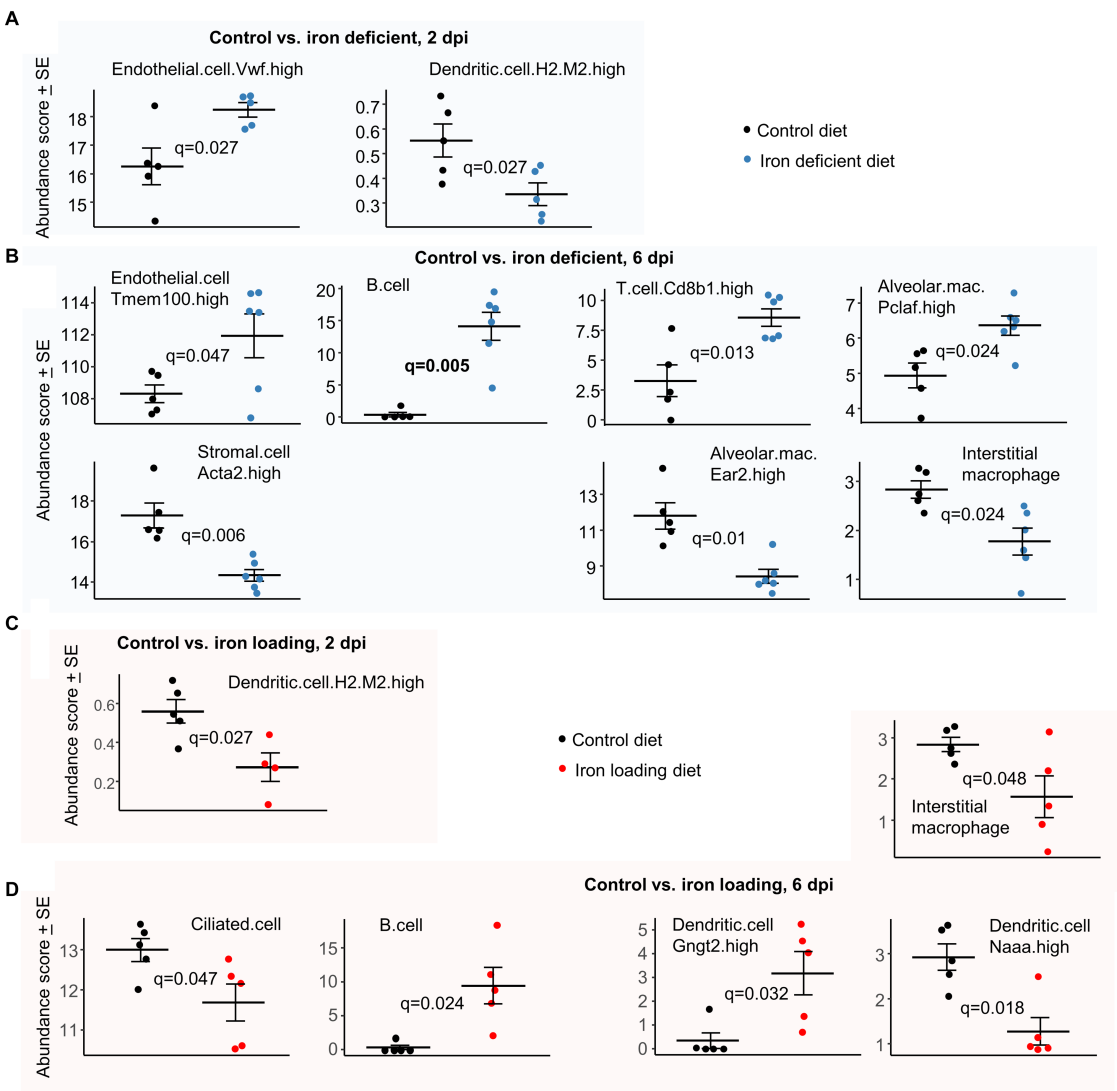
Cellular deconvolution analyses for iron deficient vs. control and iron loading vs. control diets. Relative abundances of cell types were estimated using SpatialDecon and cell-type expression matrices obtained from the NanoString Lung mouse cell atlas. **(A)** Control vs. iron deficient, 2 dpi. **(B)** Control vs. iron deficient, 6 dpi. **(C)** Control vs. iron loading, 2 dpi. **(D)** Control vs. iron loading, 6 dpi. Statistics by *t* test with FDR correction.

In iron deficient mice at 2 dpi, a significant increase in von Willebrand factor (VwF) positive endothelia cells was identified (Figure 7A). This observation is perhaps consistent with the increased extravasation annotations (Figure 3A), as endothelial VwF promotes extravasation (Petri et al., 2010; Mojzisch and Brehm, 2021).

In iron deficient mice at 6 dpi, the increase in endothelial transcriptional signatures (Figure 7B) is consistent with the increase in the eNOS signaling pathway (Figure 3B), with eNOS a key survival factor for endothelial cells during inflammation (Dimmeler and Zeiher, 1999). Stromal cells were less abundant in iron deficient mice; these cells are involved in wound repair (Jerkic et al., 2023) and their proliferation may be impaired under iron-deficient conditions (Recalcati et al., 2019). The higher B and T cell abundance scores in iron deficient mice (Figure 7B) are consistent with the BTM GSEA results (Figure 3B). Difference in mean abundance scores for B cell represents the largest and most significant (Figure 7B, *q* = 0.006) observed herein. Lung B and T cells are well described for COVID-19 (Cavalli et al., 2020; Yang et al., 2024); however, iron deficiency is generally associated with impaired B and T cell responses (Jiang et al., 2019; Wideman et al., 2023). The Interstitial macrophages are generally anti-inflammatory during disease processes (Zhou et al., 2024) and Ear2 is upregulated on alveolar macrophages under Th2 conditions (Cormier et al., 2002), so reduced abundance scores for these two cell types in iron deficient mice (Figure 7B) may be associated with the elevated Th1/Th2 ratios (Figure 3B). In contrast, proliferating alveolar macrophages (Pclaf is a proliferation marker) have a higher abundance score in iron deficient mice (Figure 7B), with these cells known to self-renew and adopt a M1 pro-inflammatory phenotype when exposed to IFNγ or TNF (Olivier et al., 2023).

In iron loaded mice at 2 dpi, only Dendritic.cell.H2.M2.high (Han et al., 2018) were identified as significantly less abundant (Figure 7C), with less infection perhaps resulting in less dendritic cell activation and/or recruitment. In iron loaded mice at 6 dpi, the abundance score for ciliated (epithelial) cells was significantly lower (Figure 7D), with these cells efficiently infected by omicron variants (Wu et al., 2023). The lower levels of infection (Figure 4C) and epithelia cell sloughing into bronchi in iron loaded mice (Figure 4J) compared with controls (Figure 1J, top), is consistent with a lower requirement for renewal and thus the reduced (transcription-based) abundance scores. The increased B cell abundance scores (Figure 7D) is consistent with BTM GSEAs (Figure 6B), with B cell abundance scores also increased in iron deficient mice (Figure 7B). A reduction in abundance scores for Interstitial macrophages at 6 dpi (Figure 7D) was similarly evident for iron deficient mice (Figure 7B), and may again be associated with the elevated Th1/Th2 ratios (Figure 6B). DCs expressing N-acylethanolamine acid amidase (Naaa) have a lower abundance score in iron loaded mice (Figure 7D). These cells are reported to play a proinflammatory role (Li et al., 2022), with reduced type I IFN responses (Figure 6B, IFNA2) perhaps contributing to their lower abundance (Tough, 2004). Gngt2 is a M1 marker (Jablonski et al., 2015) and describes a subgroup of DCs (Han et al., 2018), whose specific function has yet to be described.

Cellular deconvolution using expression matrices from the ImmGen cell family (which is based on various tissues, not just lung) further illustrates dysregulation of leukocyte subsets at 6 dpi in both iron deficient and iron loaded mice (Supplementary Figure S11).

## Discussion

We provide herein detailed comparisons of lung SARS-CoV-2 omicron XBB infection and inflammatory disease in wild-type mice that were fed an iron deficient vs. a control diet, and mice fed an iron loading vs. control diet. In iron deficient and iron loaded mice, viral loads were either not significantly affected or were mildly (≈ 0.6 log_10_), but significantly, reduced in lungs or nasal turbinates (Figure 4C, Supplementary Figure S2A). This strongly argues that the effectiveness of the host’s anti-viral SARS-CoV-2 responses was not significantly compromised by altered iron status. Modulation/dysregulation of immune responses by iron deficiency and overload is well described in various setting (Haschka et al., 2021; Mu et al., 2021), and was also clearly evident herein (Figures 3, 6, 7; Supplementary Figure S11). However, this led neither to impaired ability to control the virus nor to overtly more severe lung histopathology.

Pleiotropic outcomes of SARS-CoV-2 infections in iron deficient and iron overloaded mice might be envisaged, given the complexity of iron regulation (Galy et al., 2024), the modulation of iron biomarkers and homeostasis during SARS-CoV-2 infections (Dahan et al., 2020; Taneri et al., 2020; Onur et al., 2021; Tojo et al., 2021; Wojciechowska et al., 2021; Bastin et al., 2022; Hegelund et al., 2022; Sana and Avneesh, 2022; Aslan et al., 2023; Naidu et al., 2023), and the robust pro-inflammatory responses associated with SARS-CoV-2 infections (Bishop et al., 2022; Dharra et al., 2023; Olivier et al., 2023; Bishop et al., 2024). However, perhaps unexpected was the increase in Th1/Th2 ratios and B cell signatures in both iron deficient and iron load mice. This observation is reminiscent of the commonly illustrated U-shaped relationships for iron status. For instance, both iron deficit and iron excess leads to impaired or dysregulated maternal immunity during pregnancy (Dewey and Oaks, 2017). A similar U shaped relationship is also reported for maternal hemoglobin and preterm birth and ARDS (Ohuma et al., 2023). Serum iron biomarkers and COVID-19 severity also show a similar relationship (Tojo et al., 2021). B cell responses are also often impaired during iron deficiency (Ni et al., 2022), with IgM responses also reported to be blunted in iron loaded mice (Omara and Blakley, 1994). However, the effect of iron deficiency and overload on adaptive T cell responses would appear to be quite diverse and setting dependent (Walker and Walker, 2000; Kuvibidila et al., 2012; Reuben et al., 2017; Roth-Walter et al., 2017; Ni et al., 2022; Roth-Walter, 2022). In addition, in humans early Th1 responses have been associated with protection against severe COVID-19 (Garcia-Gasalla et al., 2022; Gil-Etayo et al., 2022); however, in mouse models, early pro-inflammatory signatures (2–7 dpi) tend to correlate with pathogenic “cytokine storm” profiles (Bishop et al., 2022; Bishop et al., 2024). Infection in humans usually spreads from the upper respiratory track into the lower respiratory track (lungs). This progression is not recapitulated in mice where lung infection requires direct intra-pulmonary inoculation of virus (Fumagalli et al., 2022; Rawle et al., 2021; Dumenil et al., 2023). Whether the increased Th1/Th2 ratios seen herein might be associated with beneficial or detrimental activities is thus debatable.

Perhaps surprising was the wide scale reduction in stress responses in iron deficient mice at 6 dpi (Figure 3, XBP1, ATF4, ROS). The most dominant of these (by z score) was XBP1, which is associated with the endoplasmic reticulum (ER) unfolded protein response (UPR). This pathway is activated by SARS-CoV-2 infection, promotes viral replication in epithelial cells, and is associated with induction of proinflammatory responses (Fernández et al., 2024a; Fernández et al., 2024b). Why this response is blunted in iron deficient mice may be related to the requirement for iron and heme effectors and binding proteins for Ire1 clustering, a process that lies immediately upstream of XBP1 activation (Cohen et al., 2017). ATF4 is triggered by PERK, another sensor of ER stress and mediator of the UPR (Davies et al., 2024). Similarly, as iron is an important component of ROS-generating enzymes (Ying et al., 2021), iron deficiency may reduce ROS production capacity in this setting.

A limitation of this study is that we have not investigated the responses over the long-term, with a role for iron status in increasing the severity of long-COVID suggested by several studies (Sonnweber et al., 2022; Wu et al., 2023; Hanson et al., 2024). However, whether mouse models (Choi et al., 2024) can faithfully recapitulate pathological or immunopathological features of human long-COVID remains to be established (Hofer, 2022), with underlying co-morbidities (Russell et al., 2023) clearly absent in genetically identical, specific pathogen free, laboratory mice. We have also not provided insights into the sizable range of co-morbidities that can give rise to anemia or iron overload (Hsu et al., 2022; Hess et al., 2023; Sohal and Kowdley, 2024), and how these would affect COVID-19; however, this would constitute a considerable undertaking. For instance, we have not studied the ‘homeostatic iron regulator’ deficient (*Hfe*^−/−^) mouse for mouse model of hereditary hemochromatosis (Albalat et al., 2021). A counter rationale for *Hfe*^−/−^ mouse studies is that compelling evidence for *Hfe* mutations affecting COVID-19 patient outcomes has yet to emerge (Ristić et al., 2023). We have also used herein a mouse model of relatively mild disease, as distinct from the more severe model involving infections of K18-hACE2 mice with original strain isolates. However, the latter is complicated by early mortality associated with fulminant brain infections (Dumenil et al., 2023), which are generally not a feature of human disease (Stewart et al., 2023). Thus, whether iron status would influence severe lung infection and disease is not addressed in the current study. Lastly, we have not investigated changes in iron distributions and their immunological consequences over time in different tissues (e.g., lungs, liver, spleen, lymph nodes) (Giorgi et al., 2015; Ali et al., 2020), primarily as there were no overt detrimental outcomes that would focus such studies.

In conclusion, the current study of iron deficient vs. control and iron loaded vs. control SARS-CoV-2 infected mice, finds modest transcriptional changes indicating a range of inflammatory response modulations, but no significant histopathologically detectable disease exacerbations. Some human studies have also failed to find a significant association between iron status and severity of acute COVID-19 (Achan et al., 2022; Marhaeni et al., 2023; Ristić et al., 2023). This is not to say that all diseases or conditions that lead to iron deficiency or overload are similarly benign, as they may indicate co-morbidities that can promote COVID-19 severity (Bhalla et al., 2021; Abu-Ismail et al., 2023).

## Materials and methods

### Ethics statements and regulatory compliance

Collection of nasal swabs from consented COVID-19 patients was approved by the University of Queensland HREC (2022/HE001492). All mouse work was conducted in accordance with the Australian code for the care and use of animals for scientific purposes (National Health and Medical Research Council, Australia). Mouse work was approved by the QIMR Berghofer MRI Animal Ethics Committee (P3600 and P3535). All infectious SARS-CoV-2 work was conducted in the BioSafety Level 3 (PC3) facility at the QIMR Berghofer MRI (Department of Agriculture, Fisheries and Forestry, certification Q2326 and Office of the Gene Technology Regulator certification 3,445). Mice were euthanized using carbon dioxide.

### Iron diet modifications

Male C57BL/6 J mice were bred in-house at the QIMR Berghofer MRI animal facility and were held under standard animal house conditions [for details see (Yan et al., 2022)]. Breeding pairs were maintained on standard rodent pellet diet (120 mg/kg iron; Norco Stockfeed, Lismore, Australia). Mice were allowed unlimited access to food and water at all times. *Iron deficient diet*. Three-week old mice were weaned onto an iron deficient diet based on AIN93G (~1 mg/kg iron, Specialty Feeds, Glen Forrest, Australia). This iron deficient diet produces a mild to moderate anemia (Mirciov et al., 2017; Zakrzewski et al., 2022). *Iron loading diet.* Three-week old mice were fed the control diet for 1 week, after which they were switched to an iron loading diet, consisting of the iron deficient diet supplemented with 0.5% iron as carbonyl iron (Sigma, Product no. C3518). This 1 week delay in switching mice to the iron loading diet is necessary, as moving weanling mice directly onto an iron loading diet dramatically reduces growth rates. *Control diet*. The control diet comprised the aforementioned iron deficient chow supplemented with 50 mg/kg iron as ferric citrate. All mice were maintained on these diets throughout until euthanasia. The aforementioned iron modified diets represent standard and ethically acceptable procedures for generating mouse models of iron deficiency and iron loading (Santos et al., 1998; Dupic et al., 2002; Aslam et al., 2014; Pereira et al., 2015).

### Assays for iron parameters

Serum (non heme) iron and transferrin saturation levels were measured using the Iron/TIBC Reagent Kit (Pointe Scientific, Canton, MI). The volumes in the kit were adjusted to allow the assay to be performed in 96-well plates as described (Frazer et al., 2017). Liver (non heme) iron levels were assayed by colorimetric assay as described previously (Fuqua et al., 2014). *Hamp* mRNA levels were determined by RT qPCR as described (Frazer et al., 2017). Serum hepcidin levels were determined using the Hepcidin Murine-Compete™ ELISA Kit (Intrinsic Life Sciences) as per manufacturer’s instructions.

### The SARS-CoV-2 omicron XBB isolate

The XBB isolate (SARS-CoV-2_UQ01_) was voluntarily donated to the University of Queensland (Brisbane, Australia) by a deidentified adult COVID-19 patient with degree-level education via a self-collected nasopharyngeal swab. The patient provided written consent (Stewart et al., 2023; Carolin et al., 2024). The isolate was initially grown on Vero E6-TMPRSS2 cells (Amarilla et al., 2021). The isolate is XBB.1.9.2.1.4 (Pango EG.1.4), a recombinant of BA.2.10.1 and BA.2.75; sequence deposited as hCoV-19/Australia/UQ01/2023; GISAID EPI_ISL_17784860. XBB viral stocks were propagated in Vero E6 cells (Rawle et al., 2021), and were titered using CCID_50_ assays (Yan et al., 2021). Medium was checked for endotoxin (Johnson et al., 2005) and cultures for mycoplasma (MycoAlert, Lonza).

### Mouse model of SARS-CoV-2 infection and monitoring of disease

Mice received intrapulmonary infections delivered via the intranasal route with 5 × 10^4^ CCID_50_ of XBB in 50 μL RPMI 1640 whilst under light anesthesia as described (Dumenil et al., 2023). Mice were weighed and monitored as described (Dumenil et al., 2023; Stewart et al., 2023).

Mice were euthanized using CO_2_, lungs were removed, with the left lung fixed in formalin for histology, the right lung inferior lobe placed in RNAlater for RNA-Seq and the remaining lobes used for tissue titers determination by CCID_50_ assays using Vero E6 cells as described (Rawle et al., 2021; Dumenil et al., 2023).

### CCID50 assays

Tissue titers were determined as described (Rawle et al., 2021). Briefly, 5-fold serial dilutions of clarified tissue homogenates were applied in duplicates to Vero E6 cells in 96 well plates. After 6 days cytopathic effects were observed by inverted light microscope. The virus titer was determined by the method of Spearman and Karber; an Excel sheet is available at https://www.klinikum.uni-heidelberg.de/zentrum-fuer-infektiologie/molecular-virology/welcome/downloads.

### Immunohistochemistry

Immunohistochemistry was undertaken using the anti-SARS-CoV-2 spike protein monoclonal antibody, SCV2-1E8, as described (Morgan et al., 2023), except that the monoclonal (IgG2a) was purified using Protein A affinity chromatography and applied to sections at 4 μg/mL for 1 h.

### Histology

Lungs were fixed in 10% formalin, embedded in paraffin, and sections stained with H&E (Sigma Aldrich). Slides were scanned using Aperio AT Turbo (Aperio, Vista, CA, United States). Quantitation of white space in scanned images of H&E stained lung parenchyma (with areas greater than ≈100 μm set as a threshold) was undertaken using PixelClassifierTools in QuPath v0.3.2 (Bankhead et al., 2017), and provides an approximate measure of lung consolidation (Dumenil et al., 2023). Scanned H&E stained whole lung sections were analyzed by Aperio Positive Pixel Count Algorithm (Leica Biosystems) to generate nuclear (strong purple staining) over cytoplasmic (total red staining) pixel count ratios, providing an approximate measure of leukocyte infiltration (Prow et al., 2019; Dumenil et al., 2023).

All H&E stained whole lung sections were scanned and .svs files examined by a trained European board-certified veterinary pathologist using Qu-Path (v 0.5.1). Lung lesions were scored using 6 criteria. Emphysema was scored; 0 = no lesion, 1 = dilated and coalescent alveoli, 2 = “bullae” in the parenchyma. Bronchial epithelium damage was score; 0 = no lesion, 1 = small clusters of necrotic epithelial cells, 2 = scattered foci of epithelial degeneration with layer architecture partial loss, 3 = focal complete epithelial loss. Bronchial content was scored; 0 = empty lumen; 1 = presence of a small amount of material; 2 = partial obliteration; 3 = complete occlusion. Vascular wall changes were scored; 0 = no lesion, 1 = leukostasis, 2 = focal wall damages (including leukocytoclasis), 3 = transmural vessel wall alteration and/or vascular lumen obliteration. Perivascular edema was scored; 0 = no lesion, 1 = focal mild edema, 2 = extended marked edema with lymphoid vessel dilatation. Peribronchial/perivascular cuffing was scored; 0 = no lesion; 1 = focal inflammatory cell infiltration; 2 = circumferential inflammatory cell infiltration, 3 = coalescing inflammatory cell infiltration between bronchi and vessels. A total cumulative score was then calculated by summing all 6 parameter scores for each mouse (range 0 to 16).

### RNA-Seq and bioinformatics

RNA-Seq (Illumina Nextseq 2000 platform generating 75 bp paired end reads) and bioinformatics was undertaken as described (Bishop et al., 2022; Bishop et al., 2024). Raw sequencing data (fastq files) have been deposited in the NCBI SRA, BioProject: PRJNA1102925 and are publicly available. Mean quality scores were above Q20 for all samples. Mouse RNA-Seq reads were aligned to a combined mouse (GRCm39, version M27) and SARS-CoV-2 BA.5 reference genome (Stewart et al., 2023) using STAR aligner. Viral read counts were generated using Samtools v1.16. RSEM v1.3.1 was used to generate expected counts for host genes. EdgeR was then used to generate TMM normalized count matrices, with a separate count matrix generated for iron deficient vs. control and iron loaded vs. control. Differentially expressed genes were identified using EdgeR using a FDR cut-off of 0.05.

Pathway analyses were performed using host DEGs and Ingenuity Pathway Analysis (IPA, v84978992) (QIAGEN), which provides Canonical pathways, Up-Stream Regulators (USR) and Diseases or Functions features as described (Dumenil et al., 2023; Bishop et al., 2024). Annotations without z scores or with significance (q or p) below 0.05 were removed.

Gene Set Enrichment Analyses (GSEAs) were undertaken using GSEA v4.1.0 with gene sets provided in MSigDB (≈ 45,000 gene sets) and in the Blood Transcription Modules (Li et al., 2014), and gene lists ranked by log_2_ fold-change. Relative abundances of cell types were estimated in R v4.1.0 from TMM normalized RSEM count matrices using SpatialDecon v1.4.3 (Danaher et al., 2022) and cell-type expression matrices obtained from the Lung mouse cell atlas^1^ and the ImmGen cell family.^2^ Statistics were undertaken by *t* tests with False Discovery Rate corrections using the Benjamini-Hochberg method (q).

### Statistics

The *t*-test was used if the difference in variances was <4 fold, skewness was > − 2 and kurtosis was <2. The *t* test significance and variance were determined using Microsoft Excel. Skewness and kurtosis were determined using IBM SPSS Statistics for Windows v19.0 (IBM Corp., Armonk, NY, United States). Otherwise, the non-parametric Kolmogorov–Smirnov exact test was performed using GraphPad Prism 10.

## Data Availability

The datasets presented in this study can be found in online repositories. The names of the repository/repositories and accession number(s) can be found in the article/Supplementary material.

## Footnotes

^1^ https://github.com/Nanostring-Biostats/CellProfileLibrary/blob/master/Mouse/Adult/Lung_MCA.RData

^2^ https://github.com/Nanostring-Biostats/CellProfileLibrary/blob/master/Mouse/Adult/ImmuneAtlas_ImmGen_cellFamily.RData
