# Supplementary material for: The effects of iron deficient and high iron diets on SARS-CoV-2 lung infection and disease
Source: Front Microbiol. 2024 Sep 4;15:1441495. doi: 10.3389/fmicb.2024.1441495 (PMC11408339; doi:10.3389/fmicb.2024.1441495)
Supplement: Supplementary file 1 [file Data_Sheet_1.pdf]

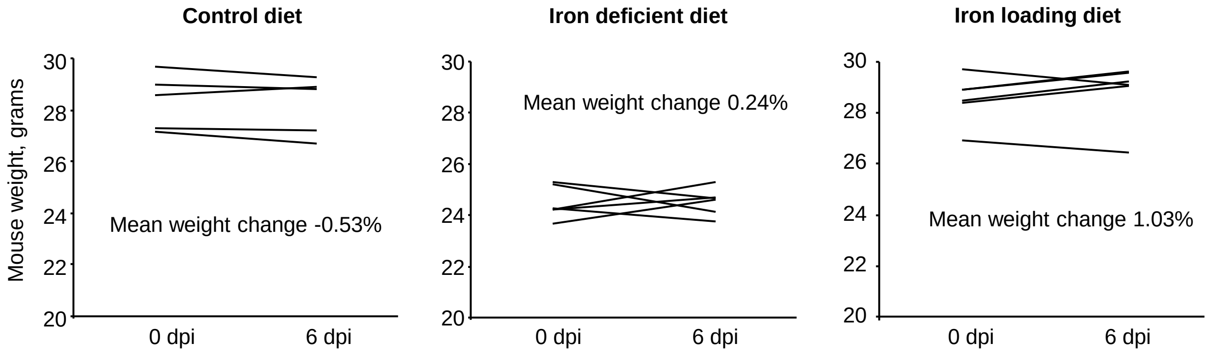

Supplementary Figure 1. Weight change post XBB infection. Mice were weighted prior to infection and prior to euthanasia at 6 dpi.

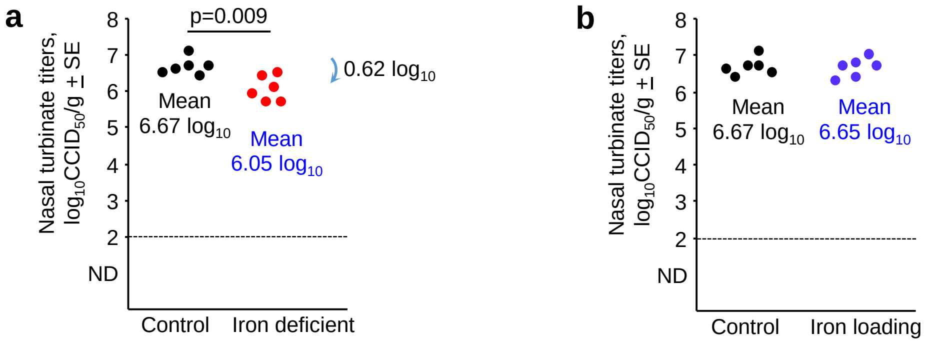

Supplementary Fig. 2. Nasal turbinated tissue titers at 2 dpi. a Titers comparing nasal turbinates from mice on control and iron deficient diets. Statistics by Mann Whitney U test. **b** Titers comparing nasal turbinates from mice on control and iron loading diets. No significant difference.

a

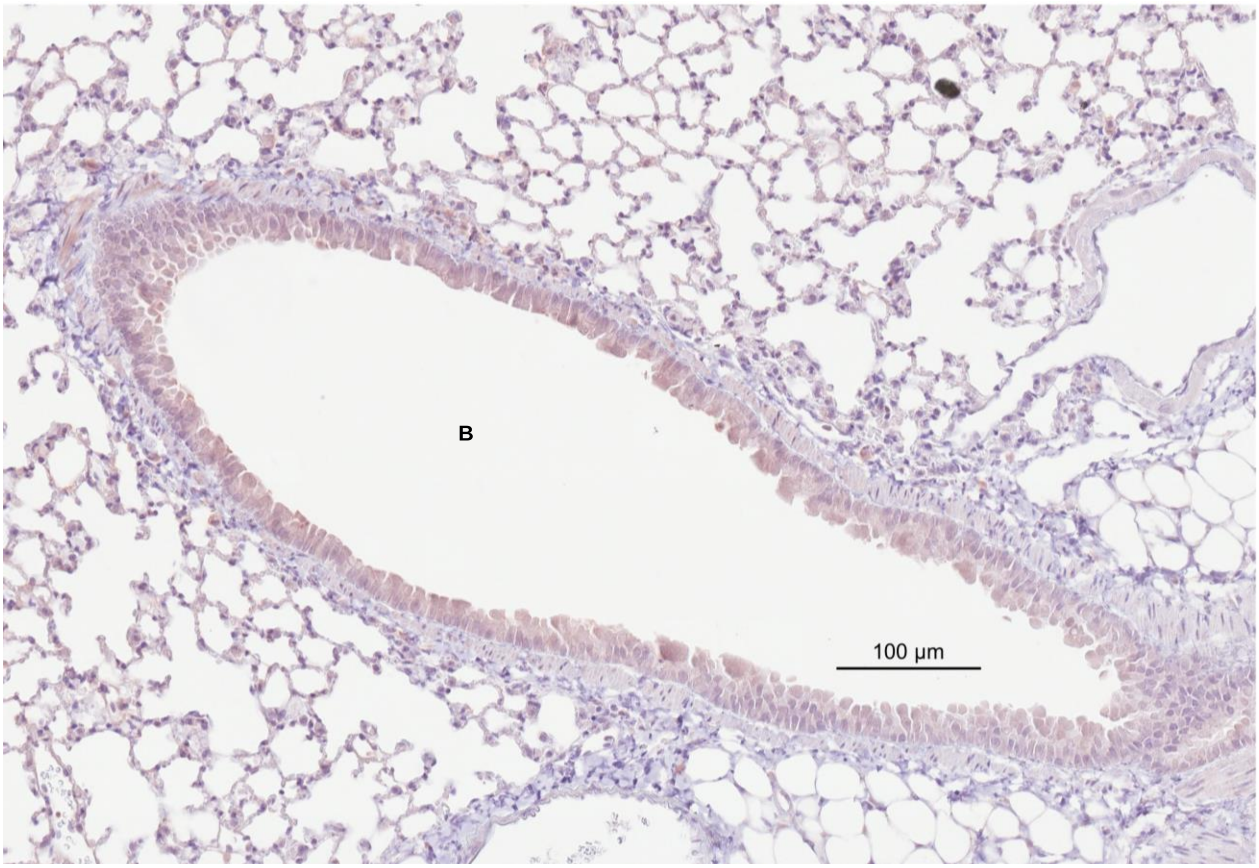

b

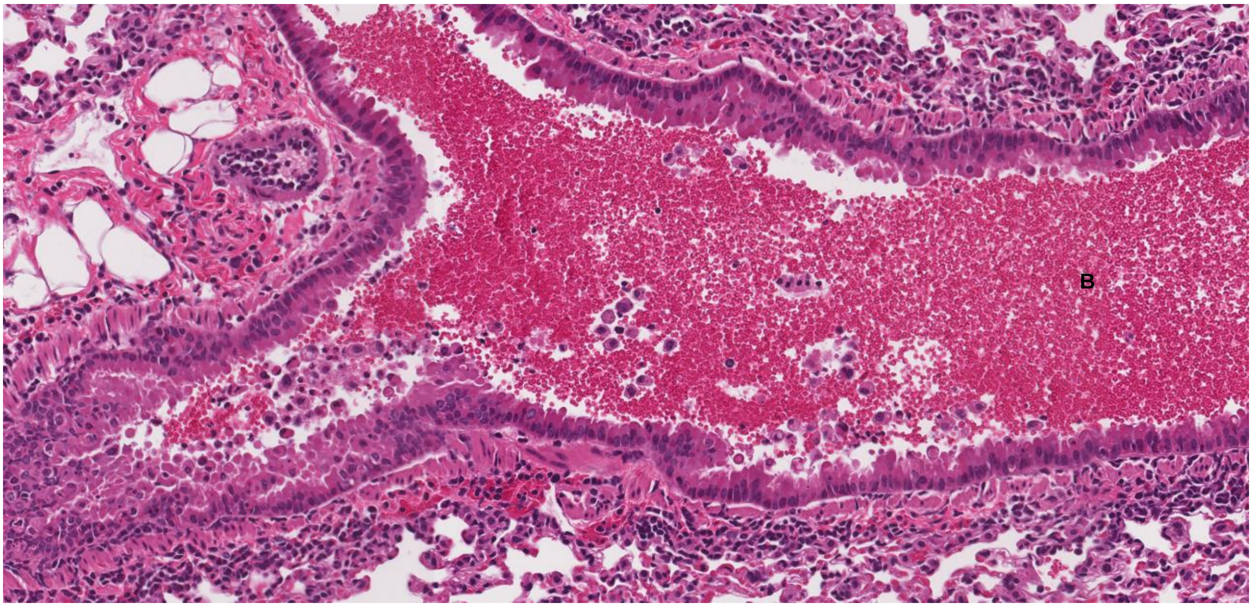

Supplementary Fig. 3. IHC control and bronchial hemorrhage. a IHC staining of an uninfected (naive) mouse lung undertaken as for Fig. 1j with the same primary and secondary antibodies. B – bronchial lumen. **b** Example of bronchial content with bronchial hemorrhage, 2 dpi control iron. B – bronchial lumen.

a

Group	Mouse ID	Diet	dpi	Emphysema (0-2)	Bronchial epithelium damage (0-3)	Bronchial content (0-3)	Vascular changes (0-3)	Perivascular edema (0-2)	Peribronchial/ perivascular cuffing (0-3)	Cumulative score (0-16)
3	1	Control	2	0	1	1	2	1	1	*6*
3	2	Control	2	0	1	2	2	2	2	*9*
3	3	Control	2	0	1	2	3	2	2	*10*
3	4	Control	2	0	2	2	3	1	3	*11*
3	5	Control	2	0	1	1	2	1	2	*7*
3	6	Control	2	0	2	1	2	1	2	*8*
			Mean	0.00	1.33	1.50	2.33	1.33	2.00	8.50
2	1	Iron deficient	2	0	2	1	3	2	2	*10*
2	2	Iron deficient	2	0	2	2	2	2	1	*9*
2	3	Iron deficient	2	1	2	2	2	2	2	*11*
2	4	Iron deficient	2	0	2	1	2	2	2	*9*
2	5	Iron deficient	2	0	1	1	0	0	1	*3*
2	6	Iron deficient	2	1	1	1	2	2	1	*8*
			Mean	0.33	1.67	1.33	1.83	1.67	1.50	8.33
6	1	Control	6	1	1	1	1	1	1	*6*
6	2	Control	6	2	2	0	1	0	2	*7*
6	3	Control	6	1	0	0	1	0	1	*3*
6	4	Control	6	0	1	1	2	1	1	*6*
6	5	Control	6	1	1	1	1	0	1	*5*
			Mean	1.00	1.00	0.60	1.20	0.40	1.20	5.40
5	1	Iron deficient	6	1	1	0	1	0	1	*4*
5	2	Iron deficient	6	1	1	0	1	0	1	*4*
5	3	Iron deficient	6	1	1	0	2	0	2	*6*
5	4	Iron deficient	6	0	1	0	2	1	1	*5*
5	5	Iron deficient	6	0	1	0	2	1	2	*6*
5	6	Iron deficient	6	0	0	0	1	1	1	*3*
			Mean	0.50	0.83	0.00	1.50	0.50	1.33	4.67

b

Group	Mouse ID	Diet	dpi	Emphysema (0-2)	Bronchial epithelium damage (0-3)	Bronchial content (0-3)	Vascular changes (0-3)	Perivascular edema (0-2)	Peribronchial /perivascular cuffing (0-3)	Cumulative score (0-16)
3	1	Control iron	2	0	1	1	2	1	1	*6*
3	2	Control iron	2	0	1	2	2	2	2	*9*
3	3	Control iron	2	0	1	2	3	2	2	*10*
3	4	Control iron	2	0	2	2	3	1	3	*11*
3	5	Control iron	2	0	1	1	2	1	2	*7*
3	6	Control iron	2	0	2	1	2	1	2	*8*
			Mean	0.00	1.33	1.50	2.33	1.33	2.00	8.50
1	1	Iron loaded	2	0	1	3	2	1	1	*8*
1	2	Iron loaded	2	1	2	1	2	2	2	*10*
1	3	Iron loaded	2	1	2	2	3	2	2	*12*
1	5	Iron loaded	2	1	1	1	2	2	2	*9*
1	6	Iron loaded	2	1	2	2	2	2	1	*10*
			Mean	0.80	1.60	1.80	2.20	1.80	1.60	9.80
6	1	Control iron	6	1	1	1	1	1	1	*6*
6	2	Control iron	6	2	2	0	1	0	2	*7*
6	3	Control iron	6	1	0	0	1	0	1	*3*
6	4	Control iron	6	0	1	1	2	1	1	*6*
6	5	Control iron	6	1	1	1	1	0	1	*5*
			Mean	1.00	1.00	0.60	1.20	0.40	1.20	5.40
4	1	Iron loaded	6	1	1	0	0	1	1	*4*
4	2	Iron loaded	6	0	0	0	1	0	1	*2*
4	3	Iron loaded	6	0	0	0	0	0	1	*1*
4	4	Iron loaded	6	1	1	0	0	0	1	*3*
4	5	Iron loaded	6	1	1	0	1	0	1	*4*
4	6	Iron loaded	6	0	0	0	1	0	0	*1*
			Mean	0.50	0.50	0.00	0.50	0.17	0.83	2.50

Supplementary Fig. 4. a Raw data for Fig. 2i. **b** Raw data for Fig. 5b.

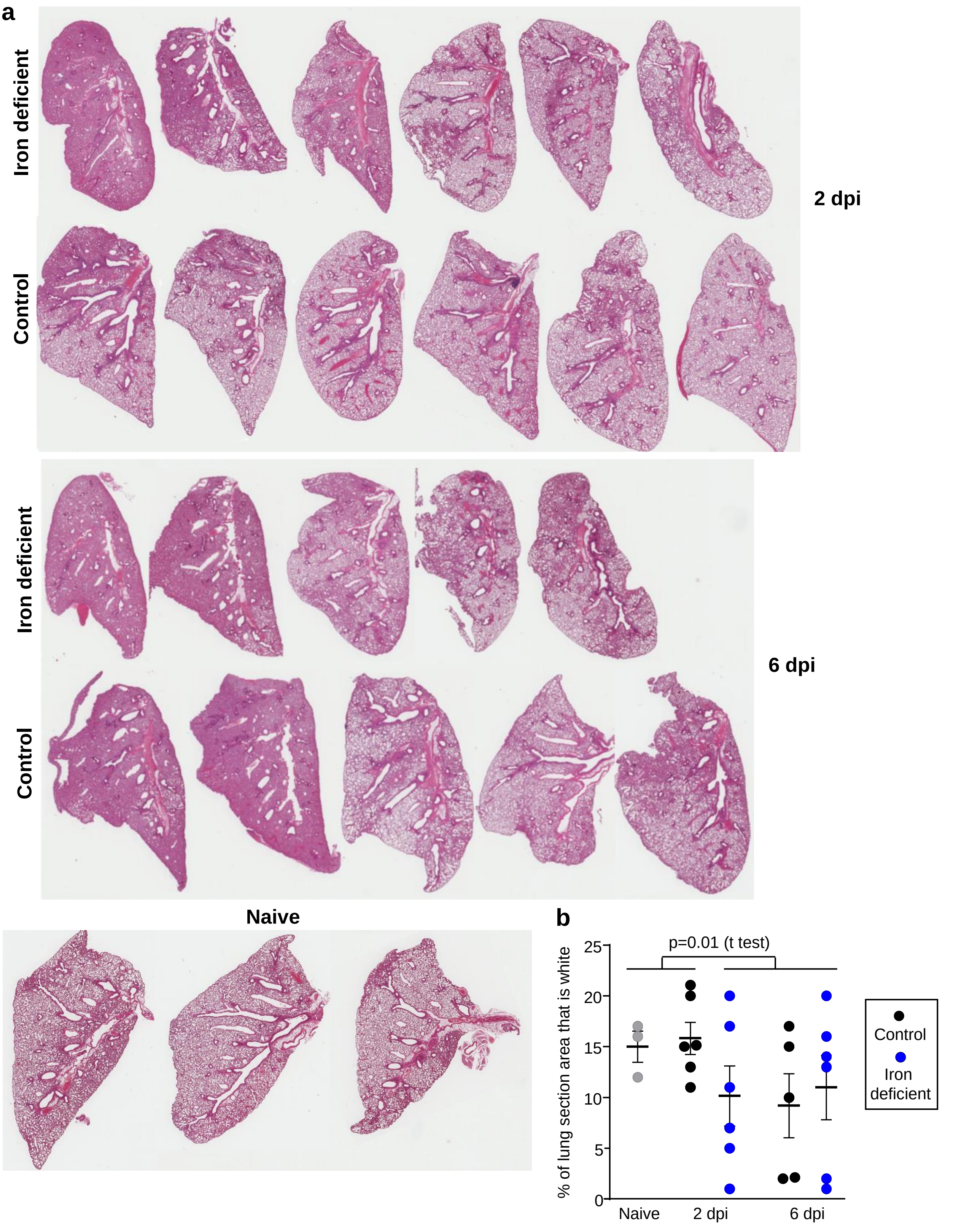

Supplementary Fig. 5. Lung consolidation iron deficient diet. a H&E of whole lung sections for the indicated groups and dpi. **b** White space analysis of a; unstained areas in the lung parenchyma as a percentage of H&E stained areas. Although white space reduction appeared to have occurred slightly earlier in some iron deficient mice (2 dpi), Control vs. Iron deficient did not reach significance at either dpi.

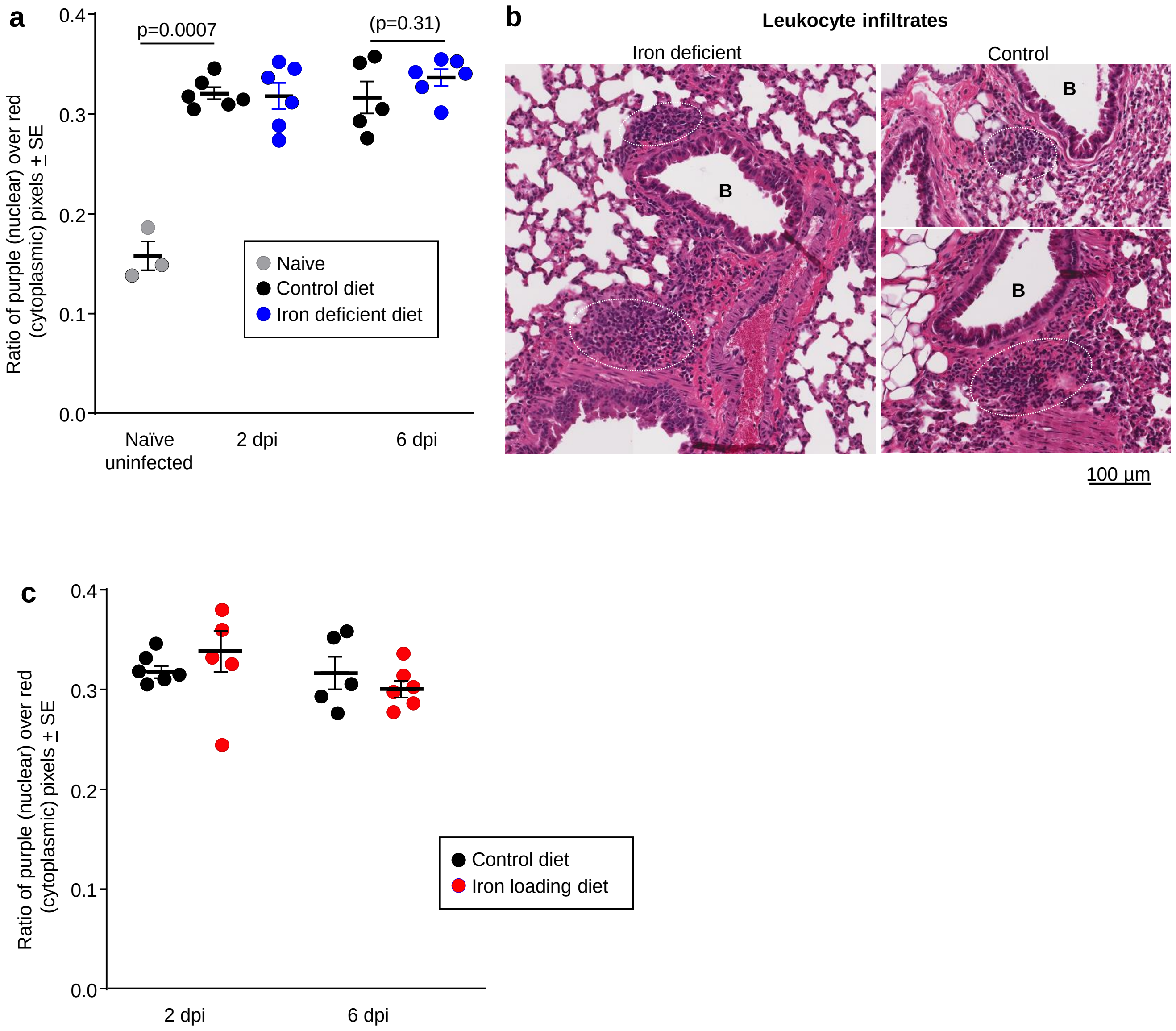

Supplementary Fig. 6. The Aperio Positive Pixel Count Algorithm (Leica Biosystems) was used to enumerate the number of strong positive purple pixels (primarily representing nuclear staining) over total red pixels (primarily representing cytoplasmic staining) in whole lung sections stained by H&E. As leukocytes have a higher nuclear to cytoplasmic ratios than resident tissue cells, this analysis provides an approximate measure of leukocyte infiltration into the tissues. Statistics by t tests.
a Ratios (nuclear/cytoplasmic) for iron deficient vs. control diets. **b** Examples H&E staining of areas with high leukocyte infiltrates (white dashed ovals), illustrating high purple/red staining ratios. B – bronchial lumen. **c** Ratios (nuclear/cytoplasmic) for iron loading vs. control diets. Control diet data is the same as that shown in a.

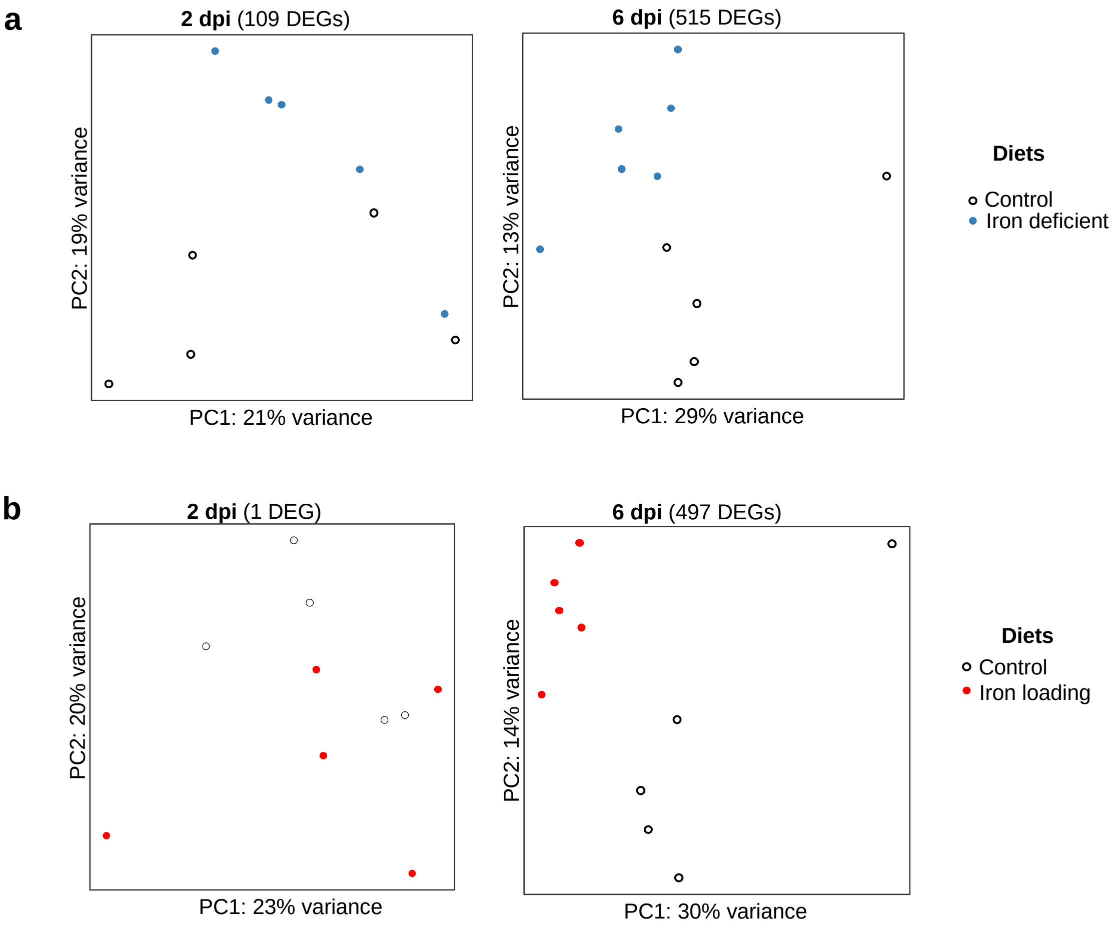

Supplementary Figure 7. PC1/PC2 plots for RNA-Seq data for lungs from XBB infected mice on 2 and 6 dpi. **a** Control vs. Iron deficient diet. **b** Control vs iron loading diet. Raw sequencing data (fastq files) have been deposited in the NCBI SRA, BioProject: PRJNA1102925 and are publicly available.

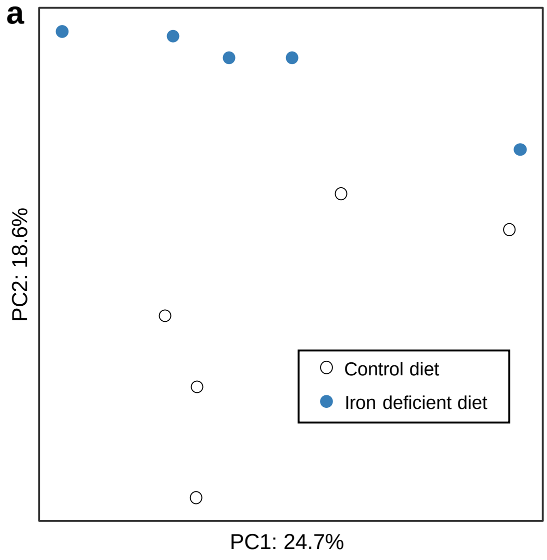

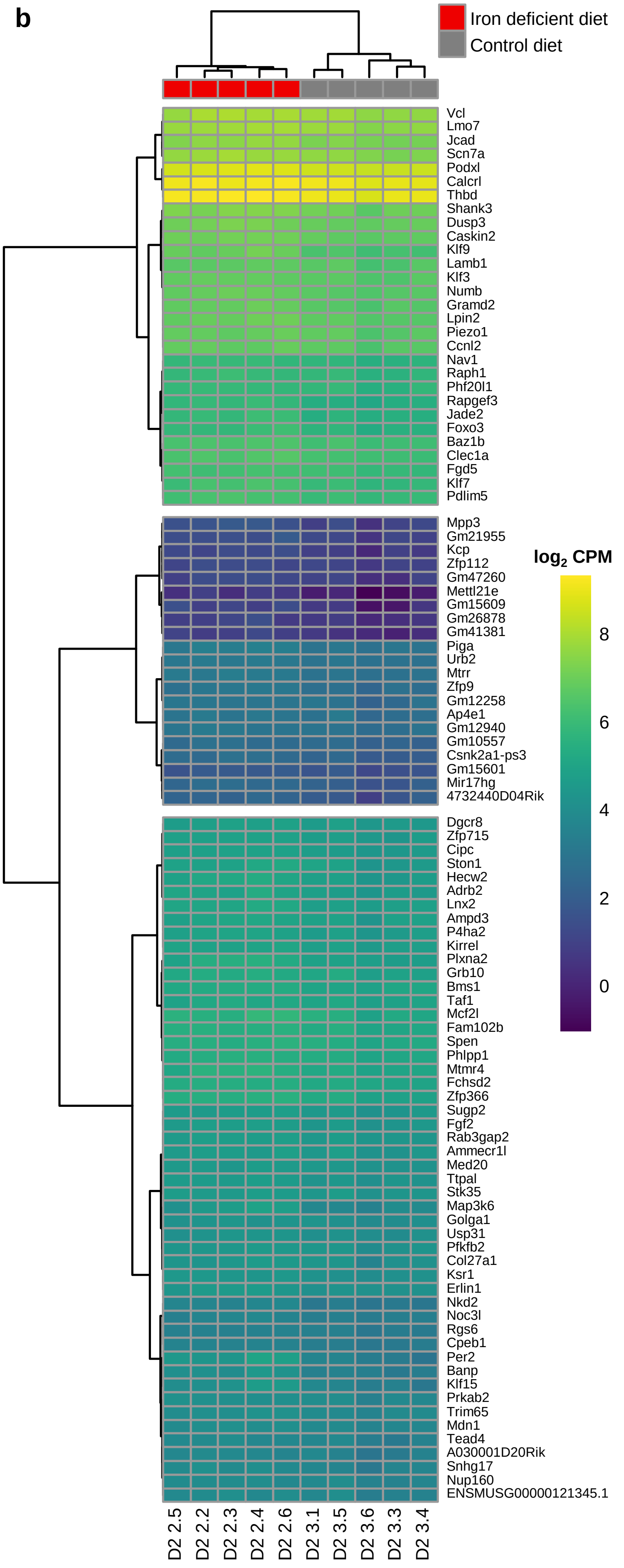

Supplementary Fig. 8. a Using the all genes list (Supplementary Table 1) variance in expression was determined for each gene. Genes were ranked by variance and the top 50% of genes showing the highest variance were used to generate the PC2/PC1 plot. This process resulted in clear segregation for the two groups along the PC2 axis. **b** The top 100 genes contributing to PC2 loading (i.e. providing the greatest contribution to the segregation between groups along the PC2 axis in a) are plotted on a heat map. The heat map shows expression in counts per million.

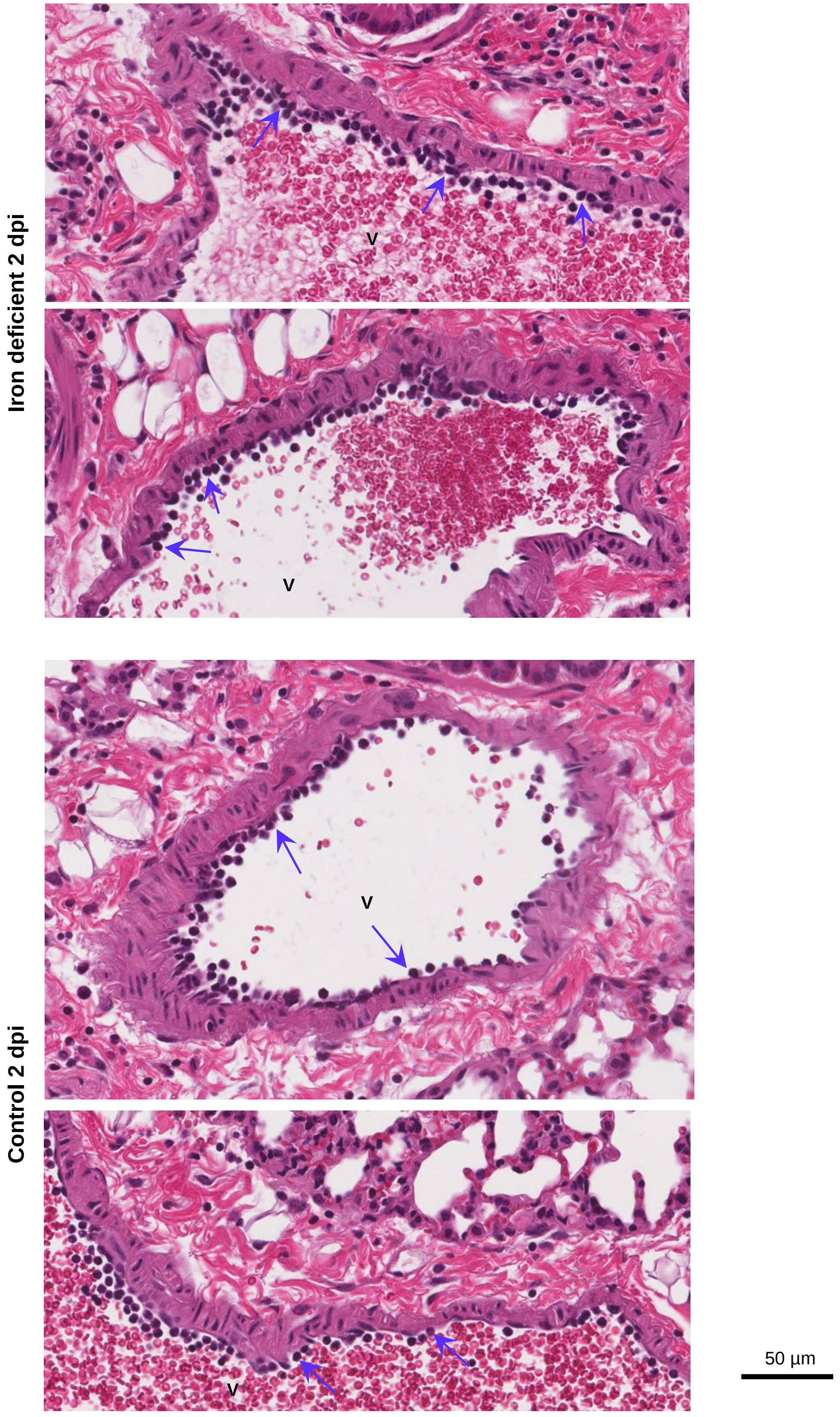

Supplementary Fig. 9. Histological features consistent with leukocyte tethering. Blue arrows indicate leukocytes closely associated with the vascular intima (leukostasis). V – vascular lumen.

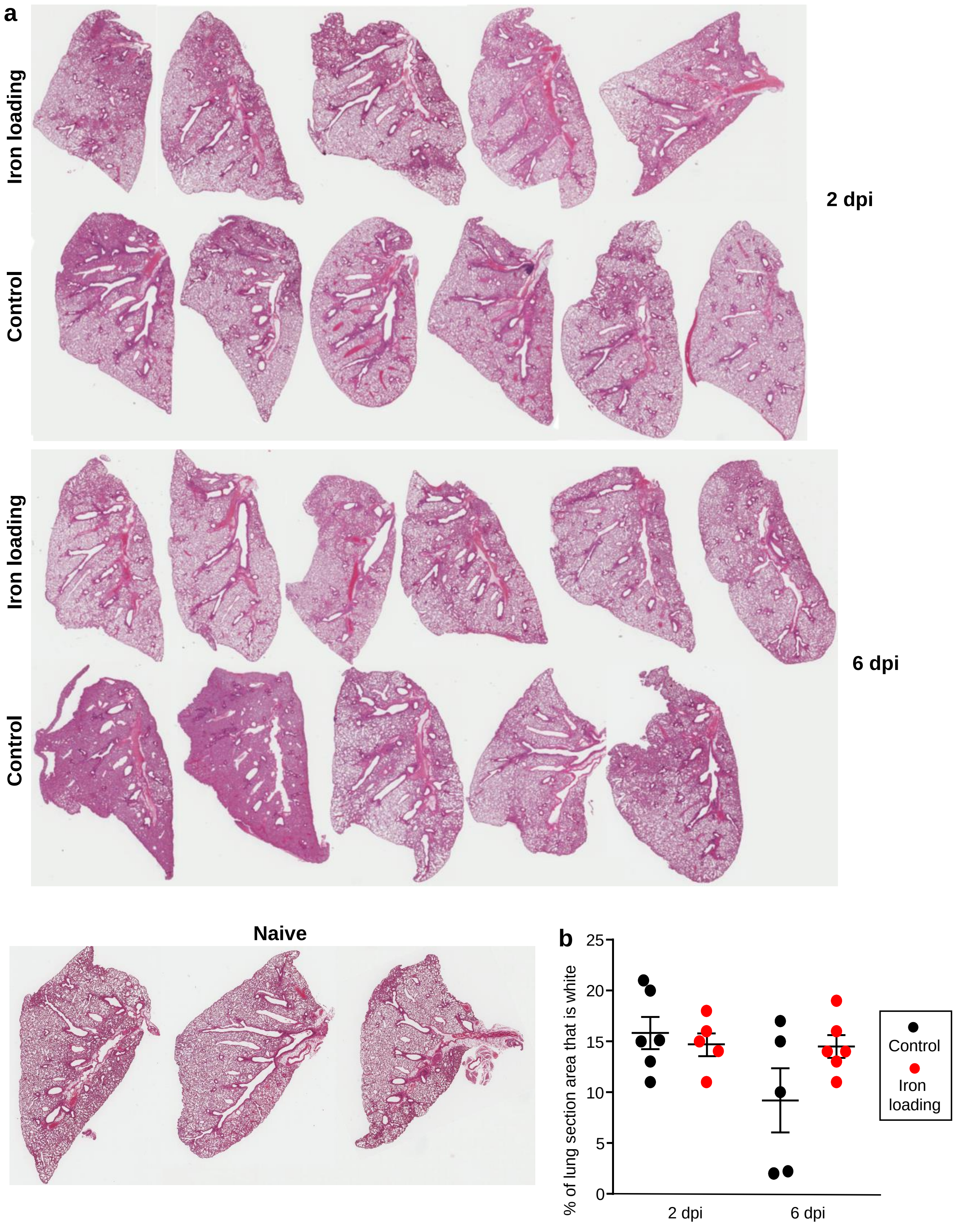

Supplementary Fig. 10. Lung consolidation in iron loaded mice. a H&E of whole lung sections for the indicated groups and dpi. **b** White space analysis of a; unstained areas in the lung parenchyma as a percentage of H&E stained areas. Although two Control mice at 6 dpi clearly showed more severe loss of white space, this was not seen in the iron loading diet group, although the differences between groups did not reach significance. Data for naïve mice is shown in Supplementary Fig. 5b.

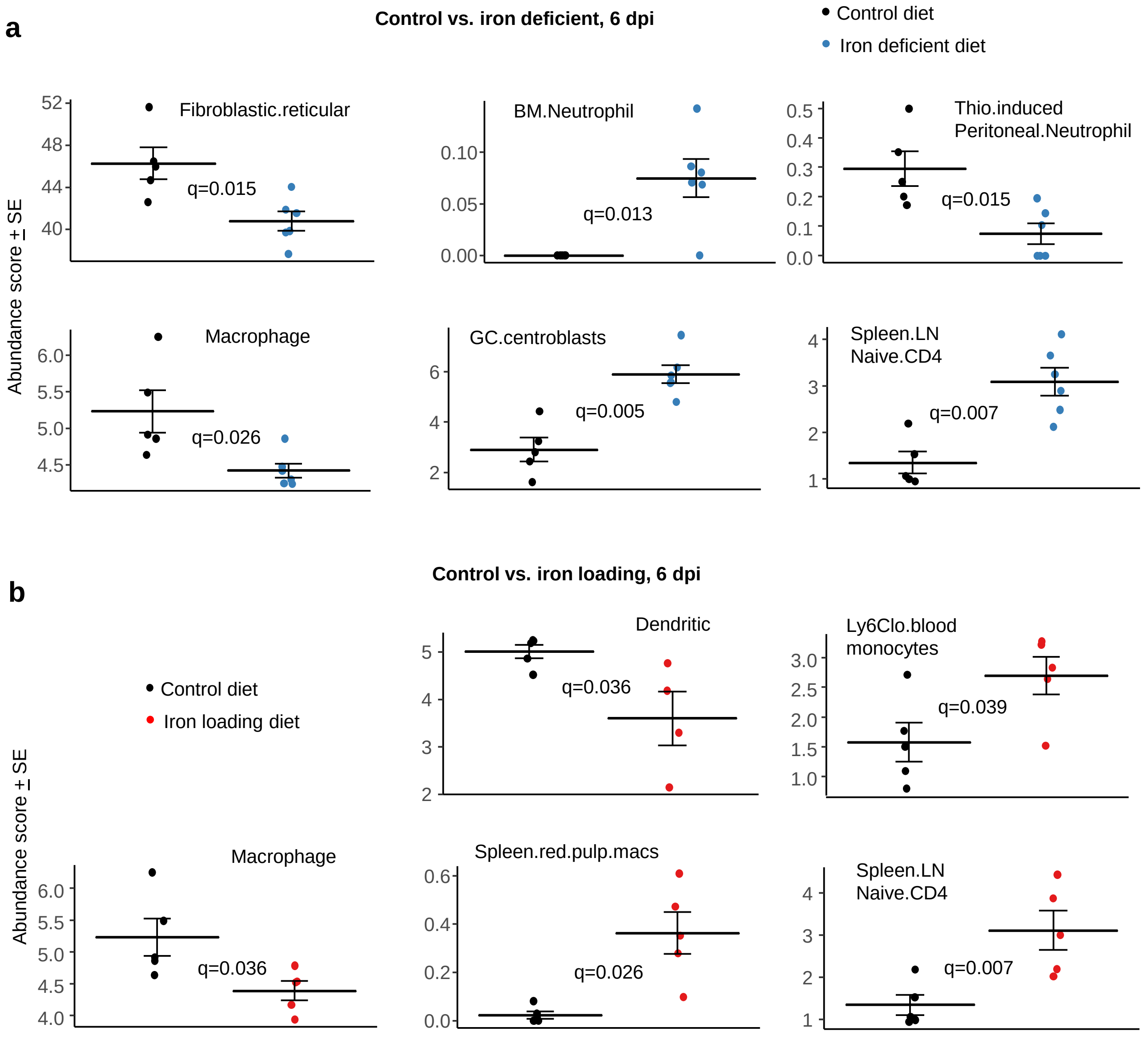

Supplementary Fig. 11. Cell deconvolution using cell-type expression matrices from the Mouse/Adult/ImmuneAtlas_ImmGen cell family. These expression matrices are not based exclusively on cells found in the lung, but are derived from cells found in various tissues. No significant differences were seen for 2 dpi. **a** Control vs. iron deficient, 6 dpi. **b** Control vs. iron loading, 6 dpi. Statistics by t test with FDR correction.
